# Trefoil factor 3 mediation of oncogenicity and chemoresistance in hepatocellular carcinoma is AKT-BCL-2 dependent

**DOI:** 10.18632/oncotarget.16950

**Published:** 2017-04-07

**Authors:** Ming-Liang You, Yi-Jun Chen, Qing-Yun Chong, Ming-Ming Wu, Vijay Pandey, Ru-Mei Chen, Liang Liu, Lan Ma, Zheng-Sheng Wu, Tao Zhu, Peter E Lobie

**Affiliations:** ^1^ Cancer Science Institute of Singapore and Department of Pharmacology, National University of Singapore, Singapore; ^2^ Hefei National Laboratory for Physical Sciences at Microscale Hefei, Anhui, China; ^3^ The CAS Key Laboratory of Innate Immunity and Chronic Disease, School of Life Sciences and Medical Center, University of Science and Technology of China, Hefei, Anhui, China; ^4^ Department of Oncology and Department of Radiology, Fudan University Shanghai Cancer Center, Fudan University, Shanghai, China; ^5^ Tsinghua Berkeley Shenzhen Institute (TBSI), Shenzhen, China; ^6^ Department of Pathology, Anhui Medical University, Hefei, Anhui, China

**Keywords:** TFF3, hepatocellular carcinoma, oncogenic, chemoresistance, cancer stem cells

## Abstract

The efficacious treatment of hepatocellular carcinoma (HCC) remains a challenge, partially being attributed to intrinsic chemoresistance. Previous reports have observed increased TFF3 expression in HCC. Herein, we investigated the functional role of TFF3 in progression of HCC, and in both intrinsic and acquired chemoresistance. TFF3 expression was observed to be upregulated in HCC and associated with poor clinicopathological features and worse patient survival outcome. Functionally, forced expression of TFF3 in HCC cell lines increased cell proliferation, cell survival, anchorage-independent and 3D matrigel growth, cell invasion and migration, and *in vivo* tumor growth. In contrast, depleted expression of TFF3 decreased the oncogenicity of HCC cells as indicated by the above parameters. Furthermore, forced expression of TFF3 decreased doxorubicin sensitivity of HCC cells, which was attributed to increased doxorubicin efflux and cancer stem cell-like behavior of Hep3B cells. In contrast, depletion of TFF3 increased doxorubicin sensitivity and decreased cancer stem cell-like behavior of Hep3B cells. Correspondingly, TFF3 expression was markedly increased in Hep3B cells with acquired doxorubicin resistance, while the depletion of TFF3 resulted in re-sensitization of the Hep3B cells to doxorubicin. The increased doxorubicin efflux and enhanced cancer stem cell-like behavior of the doxorubicin-resistant Hep3B cells was observed to be dependent on TFF3 expression. In addition, we determined that TFF3-stimulated oncogenicity and chemoresistance in HCC cells was mediated by AKT-dependent expression of BCL-2. Hence, therapeutic inhibition of TFF3 should be considered to hinder HCC progression and overcome intrinsic and acquired chemoresistance in HCC.

## INTRODUCTION

Hepatocellular carcinoma (HCC) is the predominant primary liver cancer in humans [1]. Despite the incremental improvement in its treatment over the past few decades, the mortality rate remains high as HCC is generally chemoresistant, and hence current chemotherapy has limited efficacy [2, 3]. An increasing number of studies have revealed the existence of a subpopulation of tumor-initiating cells, known as cancer stem cells (CSCs) that are responsible for tumor relapse, metastasis and radio/chemoresistance [4–6]. CSCs found in HCC (hepatic CSCs) have been reported to be associated with increased resistance to chemotherapeutic drugs and poor patient survival [7–9]. Increasing evidence has suggested that hepatic CSCs exhibit high expression level of genes involved in anti-apoptosis and drug resistance such as BCL-2 and ATP-binding cassette transporters (ABC transporters) [10, 11]. Hence, the understanding of the basic molecular mechanisms of HCC progression and hepatic CSC functions are essential to develop targeted therapeutics for HCC treatment [12, 13].

Trefoil factor 3 (TFF3) is a member of the trefoil factor family and exerts protective effects against mucosal damage in the gastrointestinal tract. TFF3 has been reported to facilitate cell migration, and inhibit apoptosis and anoikis during mucosal restitution [14]. Recent studies have reported that TFF3 expression is increased during the development and progression of human cancers, including gastric [15], breast [16, 17], colon [18], and prostate carcinomas [19] among others. TFF3 has previously been shown to stimulate survival and proliferation of mammary and prostatic carcinoma cells [16, 20]. In addition, we have previously demonstrated that TFF3 promotes metastasis and angiogenesis in mammary carcinoma [17, 21]. Importantly, increasing evidence has also supported a crucial role of TFF3 in decreasing therapeutic sensitivity and mediating therapeutic resistance. For example, our recent study has revealed that TFF3 reduces the sensitivity of prostate carcinoma cells to ionizing radiation [20]. Furthermore, several studies have also implicated functional involvement of TFF3 in resistance towards tamoxifen and aromatase inhibitors in mammary carcinomas [16, 22]. In contrast, the depletion of TFF3 has previously been shown to enhance the sensitivity of gastric cancer cells to chemotherapeutics, in particular doxorubicin [23]. TFF3 function appears to be mediated by multiple signaling pathways including mitogen-activated protein kinase (MAPK) [24], phosphatidylinositol-3-kinase-AKT (PI3K-AKT) [25, 26], signal transducer and activator of transcription 3 (STAT3) [27] and nuclear factor kappa B (NF-κB) [28]. In addition, BCL-2 is found to be an important downstream mediator of TFF3-stimulated anchorage-independent growth and anti-estrogen resistance in mammary carcinoma cells [16].

Recently, TFF3 expression has been reported to be upregulated in mouse models with spontaneous and carcinogen-induced HCC [29, 30]. TFF3 expression in HCC has also been observed to be associated with higher tumor grade [31, 32]. However, the contribution of TFF3 to the malignant progression of HCC remains unclear [33]. In this study, we report that higher TFF3 expression in HCC patient samples is associated with larger tumor size, advanced tumor grade, and a higher proliferation index as well as a poor patient survival outcome. TFF3 enhances the oncogenic behavior and CSC-like properties of HCC cells. In addition, TFF3 reduces sensitivity to doxorubicin, while increased TFF3 expression mediates acquired resistance to doxorubicin.

## RESULTS

### High TFF3 expression is associated with poor survival outcome in HCC

**Figure 1:**
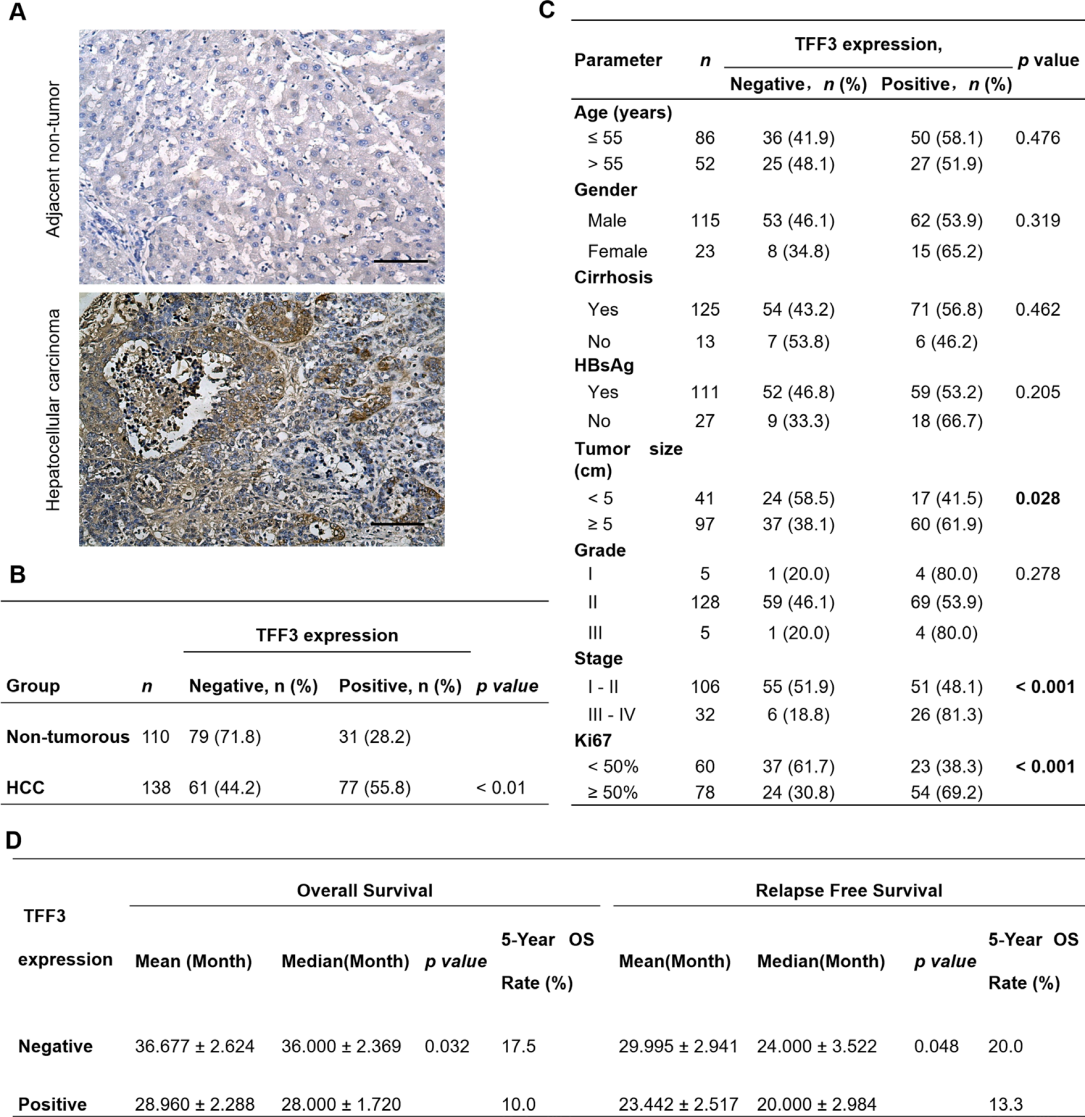
TFF3 expression correlates with poorer prognosis in HCC patients **(A)** IHC staining of TFF3 in adjacent non-tumor tissue and HCC specimens. Brown color indicates TFF3 staining. All samples were counterstained with hematoxylin and images were captured at × 100 magnification. **(B)** Statistical analysis of TFF3 expression in HCC and adjacent non-tumor tissue specimens. **(C)** Association of TFF3 expression with clinicopathological features in HCC patients. **(D)** Analysis of the significance of TFF3 expression on RFS and OS in HCC patients. OS: Overall survival; RFS: Relapse free survival.

In order to assess the clinical relevance of TFF3 expression in HCC patients, we analyzed the expression levels of TFF3 protein in adjacent non-tumor liver specimens (n=110) and HCC specimens (n=138) using immunohistochemistry (IHC). As shown in Figure 1A, higher expression levels of TFF3 protein were observed in HCC specimens, while lower expression levels were detected in the adjacent non-tumor specimens. The percentage of HCC specimens that showed positive TFF3 staining (55.8%) was approximately twice that of adjacent non-tumor liver tissues (28.2%, *p* < 0.01, Figure 1B). A positive correlation of TFF3 expression with larger tumor size (*p* < 0.05), advanced tumor stage (*p* < 0.001) and higher labeling of Ki67 (proliferation index) (*p* < 0.001) was observed (Figure 1C). On the other hand, no significant correlation of TFF3 expression was observed with patient age, cirrhosis, Hepatitis B surface antigen (HBsAg) and tumor grade. The association between TFF3 expression and HCC patient survival was assessed by using Kaplan-Meier survival analyses. As shown in Figure 1D, HCC patients with high expression levels of TFF3 exhibited a significantly shorter relapse-free and overall survival (mean and median) compared with patients expressing low levels of TFF3 protein in their tumors (*p* < 0.05). These results indicate a significant correlation between TFF3 expression and poor survival outcome in patients with HCC.

### Forced expression of TFF3 promotes oncogenicity of HCC Cells

**Figure 2:**
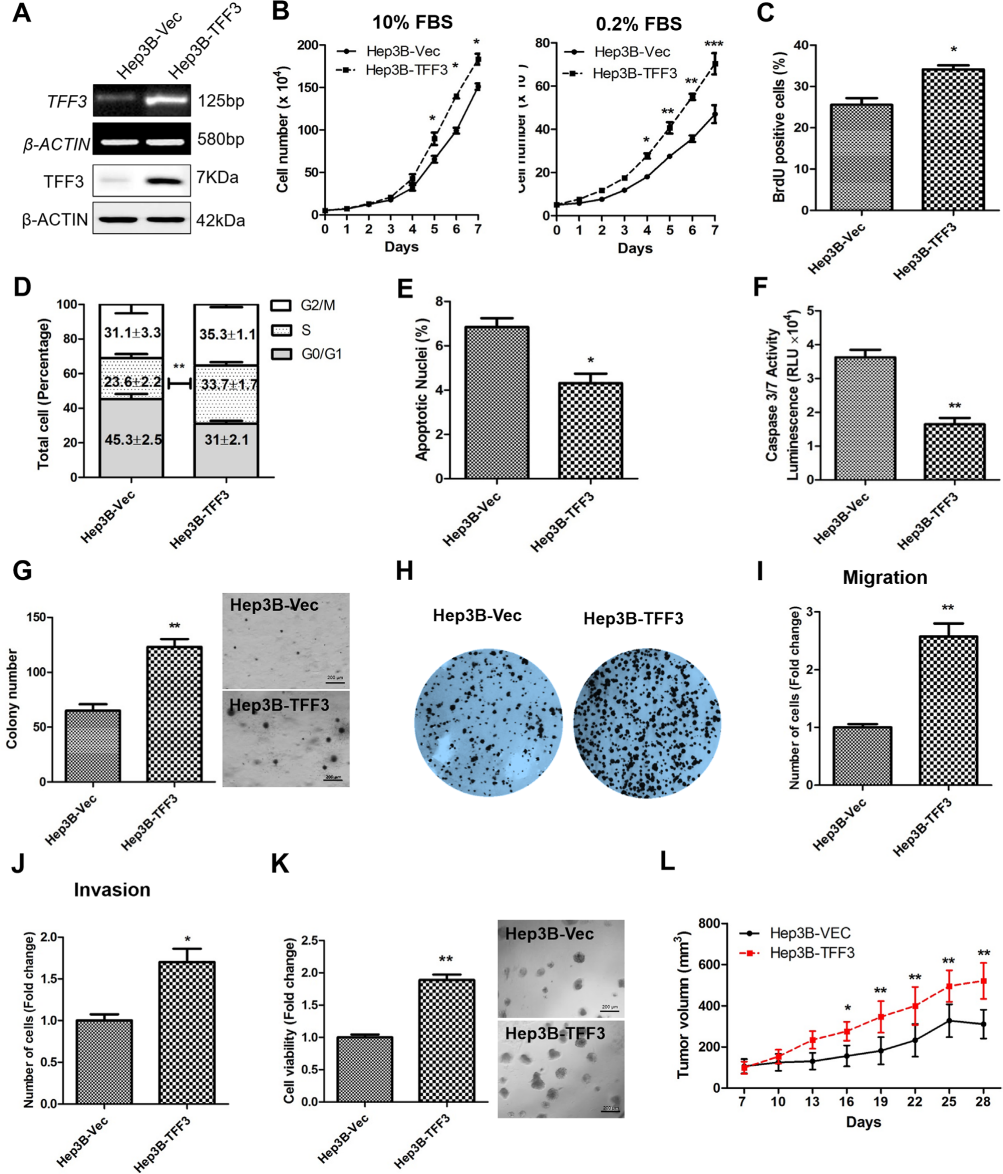
Forced expression of TFF3 promotes oncogenicity in Hep3B cells Hep3B cells were stably transfected with an expression vector containing the TFF3 gene (designated Hep3B-TFF3) or pIRESneo3 vector alone (Hep3B-Vec). **(A)** Detection of TFF3 expression with RT-PCR and western blot, β-ACTIN was used as input control. **(B)** Total cell number counting in DMEM media supplemented with 10% or 0.2% FBS over 7 days. **(C)** BrdU incorporation assay. **(D)** Cell cycle analysis. **(E)** Apoptosis assay. Percentage of apoptotic nuclei after 24h serum deprivation are shown in the histogram. **(F)** Caspase 3/7 activity after 24h serum deprivation. **(G)** Soft agar colony formation. Colony numbers are shown in the histogram. **(H)** Foci formation. **(I)** Cell migration assay. **(J)** Cell invasion assay. Number of cells penetrating the transwell membrane. **(K)** 3D Matrigel growth. Cell viability is shown in the histogram. **(L)** *in vivo* tumor formation. Tumor volumes were measured every 3 days until 6 weeks. Data were expressed as mean ±S.E.M. *, *p* < 0.05; **, *p* < 0.01; and ***, *p* < 0.001.

TFF3 mRNA and protein expression were determined in 7 HCC cell lines and the LO2 normal liver cell line. TFF3 mRNA and protein expression were observed in four of the cell lines: Huh7, Hep3B, HepG2, and PLC\PRF\5 (Supplementary Figure 1A). Based on these TFF3 expression profiles, Hep3B and Huh7 cell lines with forced expression of TFF3 were generated to investigate the functional consequences of increased TFF3 expression. Semi-quantitative RT-PCR analysis and western blot demonstrated that Hep3B-Vec cells express low levels of endogenous *TFF3* mRNA and protein. Hep3B-TFF3 cells exhibited elevated levels of TFF3 expression compared with the corresponding control Hep3B-Vec cells (Figure 2A).

The effect of TFF3 expression on HCC total cell number was examined over 7 days (Figure 2B). Hep3B-TFF3 cells exhibited a significantly higher cell number than the corresponding Hep3B-Vec cells in medium supplemented with 10% FBS and in serum-reduced medium (0.2% serum). An increase in cell number can be attributed to an increase in cell proliferation and/or decrease in cell death [34]. HCC cell proliferation was investigated by using BrdU incorporation assay and cell cycle analysis. Hep3B-TFF3 cells exhibited a higher BrdU incorporation compared with Hep3B-Vec cells (Figure 2C). Also, cell cycle analysis recorded an increase in the S-phase fraction in Hep3B-TFF3 cells (33.7 %) compared with Hep3B-Vec cells (23.6 %) (Figure 2D). These results indicate that the increased proliferation in Hep3B-TFF3 cells is at least in part due to an increase in the entry of cells to the S-phase of cell cycle. In addition, Hoechst 33342 staining (apoptotic nuclei) and Caspase 3/7 activity were measured to determine cell apoptosis in Hep3B-TFF3 and Hep3B-Vec cells. Caspase 3/7 has been shown to initiate apoptotic DNA fragmentation, which is a reliable indicator for cell apoptosis [35]. In serum-depleted conditions, forced expression of TFF3 significantly decreased apoptotic cell death in Hep3B cells, as indicated by less apoptotic nuclei in Hep3B-TFF3 cells compared with Hep3B-Vec cells (Figure 2E, *p* < 0.05). These observations were consistent with the results obtained by measuring caspase 3/7 activity, which was significantly reduced in Hep3B-TFF3 cells (Figure 2F, *p* < 0.05). Thus, besides promoting cell proliferation, TFF3 also functions as a survival factor in HCC cells.

During the process of oncogenesis, cells acquire the capability of growing in an anchorage-independent manner and resisting to anoikis [36]. In order to examine and assess this feature of oncogenic transformation *in vitro*, cancer cells were cultured in semi-solid soft agar that prevents cells from attaching to substrate. It was observed that forced expression of TFF3 dramatically increased cell anchorage-independent growth as shown in Figure 2G, whereby Hep3B-TFF3 cells formed larger and more cell colonies in soft agar compared with Hep3B-Vec cells. Furthermore, in the foci formation assay, Hep3B-TFF3 cells formed a greater number of colonies with increased size (Figure 2H), suggesting that forced expression of TFF3 increases the capacity for foci formation in Hep3B cells.

We also examined the migratory and invasive potentials of TFF3 using Transwell assays. Forced expression of TFF3 in Hep3B cells exhibited an approximate 2.5 fold increase in cell migration (Figure 2I). In addition, Hep3B-TFF3 cells demonstrated a 1.6 fold increase in invasive potential as compared to Hep3B-Vec cells (Figure 2J). The Hep3B-Vec and Hep3B-TFF3 were also cultured in growth factor-reduced Matrigel to allow three-dimensional growth in a condition that closely mimics the *in vivo* tissue environment [37]. After 9 days of growth in 4% Matrigel, Hep3B-TFF3 cells exhibited markedly higher total cell viability than Hep3B-Vec as measured using the Alamar Blue assay (Figure 2K). To verify the effect of TFF3 on other HCC cells, the same experiments were repeated in Huh7 cells and similar effects on cell behavior were observed (Supplementary Figures 1B-1K). It can be concluded that forced expression of TFF3 increases HCC cell proliferation, cell survival, anchorage independent cell growth, invasion and migration, and 3D matrigel growth.

Tumor xenografts were established by injecting Hep3B-Vec and Hep3B-TFF3 cells into the left or right flanks of nude mice (BALB/c *nu/nu* male mice). Tumors developed in all mice one week after inoculation. Consistent with *in vitro* data, forced expression of TFF3 promoted Hep3B cell–derived xenograft growth with Hep3B-TFF3 cell-derived tumors being twice the size of Hep3B-Vec cell-derived tumors after 28 days (Figure 2L).

### Depleted expression of TFF3 decreases oncogenicity of HCC Cells

**Figure 3:**
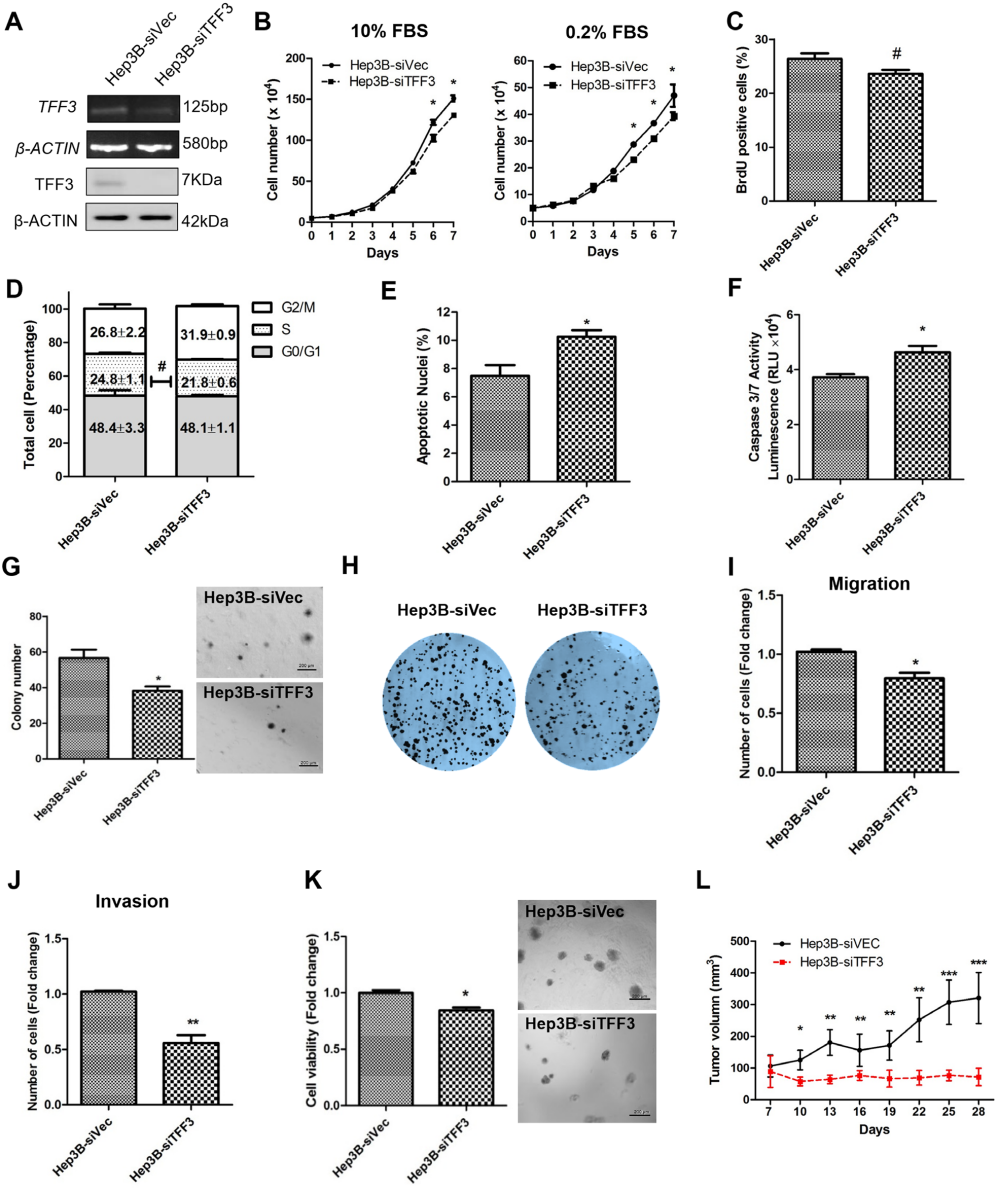
Depleted expression of TFF3 decreases oncogenicity in Hep3B cells Hep3B cells were stably transfected with an expression vector containing the TFF3 siRNA gene (designated Hep3B-siTFF3) or pSilencer vector alone (Hep3B-siVec). **(A)** Detection of TFF3 expression with RT-PCR and western blot, β-ACTIN was used as input control. **(B)** Total cell number counting in DMEM media supplemented with 10% or 0.2% FBS over 7 days. **(C)** BrdU incorporation assay. **(D)** Cell cycle analysis. **(E)** Apoptosis assay. Percentage of apoptotic nuclei after 24h serum deprivation are shown in the histogram. **(F)** Caspase 3/7 activity after 24h serum deprivation. **(G)** Soft agar colony formation. Colony numbers are shown in the histogram. **(H)** Foci formation. **(I)** Cell migration assay. **(J)** Cell invasion assay. Number of cells penetrating the transwell membrane. **(K)** 3D Matrigel growth. Cell viability is shown in the histogram. **(L)** *in vivo* tumor formation. Tumor volumes were measured every 3 days until 6 weeks. Data were expressed as mean ±S.E.M. *, *p* < 0.05; **, *p* < 0.01; and ***, *p* < 0.001; #, no significance

A Hep3B cell model with depleted TFF3 expression was generated and the resulting functional consequences were investigated. After transfection with the pSilencer-TFF3 plasmid [16], Hep3B-siTFF3 cells were observed to express lower levels of TFF3 (Figure 3A) and exhibited smaller increases in cell number in both 0.2% and 10% FBS supplemented medium (Figure 3B) as compared to its corresponding control Hep3B-siVec cells. However, BrdU incorporation results did not show a statistically significant difference between Hep3B-siTFF3 and Hep3B-siVec (Figure 3C). Also, cell cycle analysis showed only a slight decrease in the S-phase fraction of cells in Hep3B-siTFF3 cells compared with Hep3B-siVec cells (Figure 3D). More precisely, 24.8% of Hep3B-siVec cells were in S-phase compared with 21.8% of Hep3B-siTFF3 cells (no statistical significance). Nevertheless, the depletion of TFF3 increased the proportion of apoptotic nuclei and caspase 3/7 activity of Hep3B cells (Figures 3E and 3F, p < 0.05). These results indicate that a decrease in cell survival, rather than a decrease in proliferation, leads to the reduction in total cell number of Hep3B cells upon depletion of TFF3.

In the soft agar formation assay, depleted expression of TFF3 dramatically decreased cell anchorage-independent growth (Figure 3G). Hep3B-siTFF3 cells produced only an average of 37.2 colonies per well, whereas Hep3B-siVec cells produced an average of 58.5 colonies per well (*p* < 0.05). In the foci formation assay, Hep3B-siTFF3 cells formed less and smaller colonies compared to Hep3B-siVec cells (Figure 3H).

The depletion of TFF3 significantly reduced migration of Hep3B cells (Figure 3I). Furthermore, Hep3B-siTFF3 cells exhibited decreased invasive potential as compared to Hep3B-siVec cells (Figure 3J). In 3D matrigel growth assay, there was a significant decrease in cell viability in Hep3B-siTFF3 cells compared with Hep3B-siVec cells (Figure 3K). The similar effects were also observed in HepG2 cell with TFF3 depletion (Supplementary Figures 2A-2J). Thus, depletion of TFF3 reduces cell survival, anchorage independent cell growth, invasion and migration, and 3D matrigel growth of HCC cells.

Tumor xenografts derived from Hep3B-siVec and Hep3B-siTFF3 cells were similarly established in nude mice. Consistent with *in vitro* data, the depletion of TFF3 resulted in attenuated tumor growth of Hep3B-siTFF3 cell-derived xenograft as compared to Hep3B-siVec cell-derived xenograft (Figure 3L). Hence, it can be concluded that the modulation of TFF3 expression affects HCC growth *in vivo*.

### TFF3 modulates cell cycle, apoptotic and EMT-related gene expression in HCC cells

A gene expression profile was analysed using qPCR to examine the effect of forced or depleted expression of TFF3 on the relative expression of genes involved in cell cycle progression and cell survival (Table 1). Forced expression of TFF3 increased the expression of *CDK4* and *CDK2* (cyclin-dependent kinase) and their regulators *CCND1* (Cyclin D1) and *CCNE1* (Cyclin E1), which are directly involved in cell cycle regulation and are required for cell cycle G1/S transition [38, 39]. However, those genes were not significantly altered in Hep3B-siTFF3 cells, which was consistent with the cell cycle analysis results (Figure 3D). *CDKN1A* (cyclin-dependent kinase inhibitor/p21), a negative regulator of cell growth [38], was found to be reduced in Hep3B-TFF3 cells but increased in Hep3B-siTFF3 cells. Such gene expression profiles are consistent with the increase in BrdU-positive cells and S-phase population in Hep3B-TFF3 cells. TFF3-stimulated cell proliferation is attributed to the regulation of these cell cycle regulators by TFF3. These observations are consistent with a previous study that TFF3 promotes the cell cycle through the accumulation of cyclin D1 to promote cell proliferation [40].

**Table 1:** Forced/depleted expression of TFF3 alters the gene expression in Hep3B cells

Functional Gene Grouping	Gene	TFF3/VecFold Change	*p value*	siTFF3/siVecFold Change	*p value*
Cell Cycle Control & DNA Damage Repair	*CCND1*	2.67	1.73E-04	0.94	2.67E-01
	*ATM*	1.10	3.18E-03	0.93	6.45E-02
	*BRCA1*	1.35	2.60E-06	0.54	1.30E-01
	*CCNE1*	2.49	3.31E-04	0.78	7.74E-02
	*CDC25A*	1.47	2.54E-04	0.92	6.85E-02
	*CDK2*	1.41	1.99E-04	0.87	4.52E-01
	*CDK4*	3.01	4.02E-05	1.05	2.92E-03
	*CDKN1A*	0.34	1.41E-05	2.21	6.35E-02
	*CDKN2A*	0.97	3.18E-01	1.77	8.94E-03
	*CHEK2*	0.73	1.22E-04	1.55	8.55E-02
	*E2F1*	1.29	2.43E-04	0.93	1.08E-01
	*MDM2*	0.54	6.98E-03	0.99	1.57E-01
	*RB1*	0.89	3.41E-04	0.98	2.09E-03
	*S100A4*	1.14	9.99E-04	1.19	1.21E-01
	*TP53*	1.01	4.09E-04	1.00	1.20E-03
	*CDKN1B*	0.99	1.56E-04	1.30	1.59E-02
Apoptosis and Cell Senescence	*APAF1*	0.95	2.98E-03	1.40	3.96E-03
	*BCLAF1*	0.89	2.11E-03	1.00	7.36E-02
	*BAK1*	0.73	1.74E-04	7.01	3.32E-03
	*BAD*	0.53	2.33E-05	1.61	8.95E-04
	*BAX*	0.22	1.56E-02	4.08	4.32E-03
	*BCL2*	6.56	3.15E-05	0.37	5.68E-02
	*BCL2L1*	2.75	2.07E-04	0.64	1.90E-02
	*CFLAR*	0.92	3.80E-03	1.20	8.95E-04
	*CASP7*	0.44	4.62E-04	1.84	8.97E-03
	*GZMA*	1.06	4.05E-02	0.74	3.05E-01
	*HTATIP2*	0.95	8.33E-05	0.55	1.46E-04
	*TERT*	3.82	2.07E-03	0.42	1.43E-03
	*TNFRSF1A*	1.01	2.61E-03	1.11	2.30E-03
	*TNFRSF10B*	1.05	3.51E-05	1.04	1.68E-01
	*TNFRSF25*	0.97	1.45E-01	0.67	5.79E-03
EMT and stem cell	TWIST1	1.45	1.07E-01	1.04	5.93E-02
	FN1	0.62	5.77E-04	0.93	4.36E-01
	SNAIL	0.89	2.35E-01	1.36	2.15E-02
	SNAIL2	6.06	9.46E-04	0.67	2.38E-01
	CTNNA1	0.94	6.90E-03	0.61	3.83E-02
	CTNNB1	1.00	5.54E-03	2.27	1.28E-03
	VIM	0.58	5.93E-04	0.78	2.80E-01
	CDH1	0.60	2.11E-01	0.92	4.07E-01
	CDH2	0.90	1.69E-04	1.07	1.91E-01
	ZEB1	2.34	1.23E-01	0.94	4.16E-01
	ZEB2	1.76	1.03E-03	1.07	1.23E-01
	NCAM1	0.76	3.26E-02	2.00	1.78E-02
	OCLN	0.47	9.67E-04	5.34	2.11E-01
	FOXC2	2.72	3.14E-02	0.94	2.87E-01
	SALL4	4.97	1.19E-01	0.31	7.99E-03
	CSF1	3.90	2.12E-01	1.10	2.51E-01
	NOTCH1	0.82	2.96E-01	0.96	1.57E-02
	IGF1	1.38	1.17E-01	1.62	5.48E-03
	KLF4	1.31	2.86E-03	0.77	5.81E-03
	SOX2	0.93	2.62E-03	0.50	1.68E-02
	ALDH1	3.75	1.94E-03	0.38	2.00E-02
	WNT5A	0.94	3.61E-02	0.98	1.41E-01
	WNT5B	0.96	1.99E-01	1.27	9.37E-02
	NANOG	2.81	1.13E-02	0.81	3.36E-01
	*BMI1*	3.75	6.78E-02	0.34	2.70E-02
	*POU5F1*	0.91	4.81E-02	1.03	4.55E-02
	*CD24*	0.97	1.60E-02	0.36	1.35E-02
	*CD44*	3.39	3.91E-02	0.50	3.16E-02
	*CD133*	3.97	3.89E-03	0.80	3.51E-01

The qPCR experiment was performed three times and the result shown as mean ± S.E.M. of triplicates in a representative experiment.

*BCL-2*, an anti-apoptotic gene, exhibited increased mRNA expression of more than 6 fold in Hep3B-TFF3 cells and decreased mRNA expression in Hep3B-siTFF3 cells as compared to their respective control cells (Table 1). In contrast, *BAX*, a cell death mediator inducing mitochondrial damage [41], was significantly downregulated in Hep3B-TFF3 cells and upregulated in Hep3B-siTFF3 cells as compared to the control cells (Table 1). In addition, mRNA levels of several pro-apoptotic genes, *BAD*, and *CASP7* (Caspase7) were also decreased with forced expression of TFF3 and increased with depletion of TFF3 in Hep3B. Furthermore, *TERT* mRNA was found to be increased in Hep3B-TFF3 but decreased in Hep3B-siTFF3 cells as compared to their relative control cells, potentially indicative of decreased cellular senescence and enhanced cell survival [42]. These results are concordant with the decreased apoptosis observed in Hep3B-TFF3 cells.

In addition, the mRNA expression of several mesenchymal gene markers, including SNAIL2, FOXC2 and ZEB2, was observed to be increased, while that of an epithelial gene marker OCLN was decreased in Hep3B-TFF3 as compared to Hep3B-Vec cells, consistent with TFF3-stimualted invasion and migration of HCC cells. Although TFF3 was observed to stimulate EMT of HCC cells, the classic EMT gene expression pattern was not completely observed upon forced expression or depletion of TFF3 in HCC cells.

### TFF3 reduces the sensitivity of HCC cells to doxorubicin

**Figure 4:**
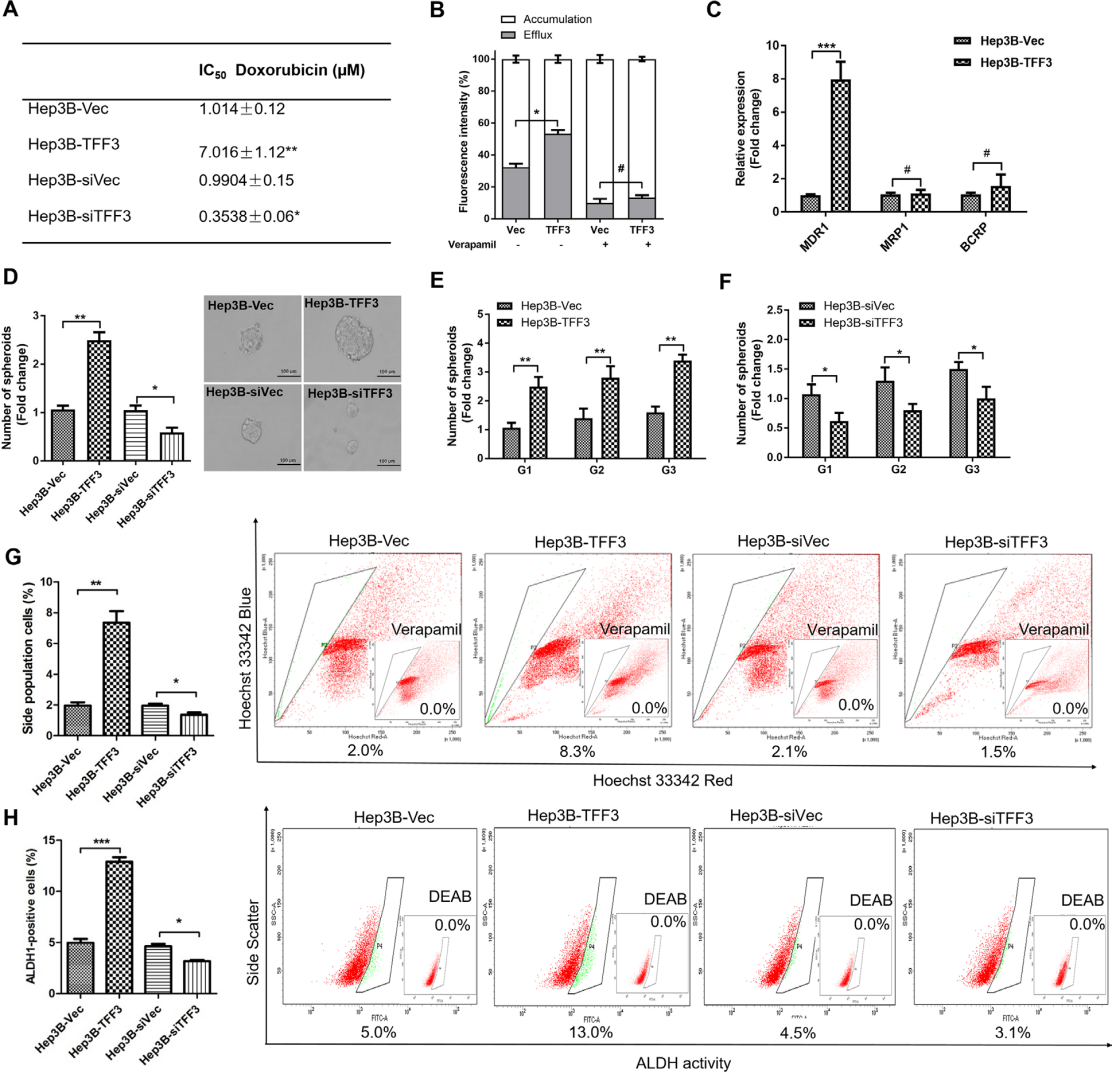
TFF3 decreases chemosensitivity and promotes CSC-like properties in HCC cells **(A)** The IC_50_ of doxorubicin in Hep3B was measured and analysed using nonlinear regression. **(B)** Efflux and accumulation of doxorubicin in Hep3B cells. **(C)** q-PCR analysis of genes involved in drug resistance. Verapamil was used to block the ABC membrane transporter from extruding the doxorubicin. **(D)** Spheroid formation. The number of spheroids formed by Hep3B-Vec, -TFF3, siVec and –siTFF3 cells is shown in the histogram. **(E)** Spheroid formation ability after passage to G3 in Hep3B-TFF3 and Hep3B-Vec. **(F)** Spheroid formation ability after passage to G3 in Hep3B-siTFF3 and Hep3B-siVec. **(G)** The percentages of side-population in Hep3B-Vec, -TFF3, siVec and –siTFF3 cells were determined by flow cytometry. Verapamil was used to inhibit the ABC membrane transporter from extruding the Hoechst 33342 dye to establish the baseline for the identification of side population cells that efflux the Hoechst 33342 dye. **(H)** The percentages of ALDH+ cells in Hep3B-Vec, -TFF3, siVec and –siTFF3 cells were analysed using flow cytometry. The cells were incubated with the ALDEFLUOR substrate to define ALDH+ cells, while DEAB, a specific inhibitor for ALDH1A1 isoform, was used to establish the baseline fluorescence. Data were expressed as mean ±S.E.M. *, *p* < 0.05; **, *p* < 0.01; and ***, *p* < 0.001; #, no significance.

Doxorubicin is a commonly used chemotherapeutic drug for the treatment of advanced HCC, and intrinsic resistance to doxorubicin is a major challenge [43]. TFF3 has previously been reported to be upregulated in liver tissue and hepatocytes following doxorubicin administration [44]. In order to investigate the possible role of TFF3 in HCC chemosensitivity, we determined the effects of forced or depleted expression of TFF3 on doxorubicin sensitivity in Hep3B cells. The half maximal inhibitory concentration (IC_50_) of doxorubicin was measured in Hep3B cells with forced or depleted expression of TFF3 and analysed using nonlinear regression, summarized in Figure 4A. Forced expression of TFF3 significantly increased the IC_50_ of doxorubicin by 7 fold (Hep3B-Vec/Hep3B-TFF3= 1.01μM/7.02μM, *p* < 0.005) in Hep3B cells. In contrast, depleted expression of TFF3 significantly decreased the IC_50_ of doxorubicin (Hep3B-siVec/Hep3B-siTFF3 = 0.99μM/0.35μM, *p* < 0.01) by 3 fold in Hep3B cells. Hence Hep3B-TFF3 cells exhibited lower chemosensitivity compared to Hep3B-Vec cells, while Hep3B-siTFF3 showed higher chemosensitivity than Hep3B-siVec cells.

It has been reported that drug resistance in cancer cells correlates with drug efflux [45]. We observed a significant increase in the efflux of doxorubicin and a significant decrease in the cellular accumulation of doxorubicin in Hep3B-TFF3 cells as compared to Hep3B-Vec cells (Figure 4B). Several ABC transporters function as drug efflux pumps which are responsible for intrinsic resistance [45, 46]. Multidrug resistance 1 gene (*MDR1*) was significantly increased in HCC cells with forced expression of TFF3. However, the other two ABC drug efflux transporters, breast cancer resistance protein (BCRP/ABCG2) and MRP1 (ABCC1), did not exhibit significant differences of expression in Hep3B-TFF3 cells versus Hep3B-Vec cells (Figure 4C). Furthermore, Verapamil, an MDR1 inhibitor, significantly decreased doxorubicin efflux and increased doxorubicin accumulation in Hep3B-Vec cells, and abrogated the enhanced doxorubicin efflux in Hep3B-TFF3 cells (Figure 4B). These results suggest that enhancement of drug efflux by TFF3 in Hep3B cells is MDR1-dependent.

### TFF3 increases cancer stem cell-like behavior in HCC cells

Previous studies have suggested that CSC-like HCC cells are the culprits for doxorubicin- and 5-FU-resistance [10], and these spheroid-forming HCC cells exhibit stem cell-like properties [47]. Hep3B cells with forced expression of TFF3 exhibited a marked increase in the formation of spheroids compared with Hep3B-Vec control cells. In contrast, Hep3B cells with depleted expression of TFF3 formed fewer spheroids compared with its Hep3B-siVec control cells (Figure 4D). In order to confirm that the increase in spheroid formation represents the progeny of individual CSC rather than the aggregation of quiescent cells, we examined the spheroid-forming ability of primary spheroid-forming cells (G1) to form second generation (G2) and third generation (G3) spheroids. The forced expression of TFF3 enhanced G2 and G3 spheroid formation, while the depletion of TFF3 reduced G2 and G3 spheroid formation in Hep3B cells (Figures 4E-4F).

Side-population (SP) cells isolated from HCC cells exhibit CSC-like properties, suggestive of a contribution of this cell population to chemoresistance [48]. Flow cytometry analysis recorded a significant increase in the proportion of SP cells in Hep3B-TFF3 cells (8.3 ± 0.4%) compared with Hep3B-Vec cells (2.0 ± 0.1%). Conversely, the percentage of SP cells was decreased in Hep3B-siTFF3 cells (1.5 ± 0.1%) compared with Hep3B-siVec cells (2.1 ± 0.2%) (Figure 4G). Previous studies have also suggested the utility of aldehyde dehydrogenase 1 (ALDH1) to identify liver stem cells [49]. To determine whether TFF3 modulated the ALDH1-positive (ALDH1+) cell population in HCC cells, we measured the percentage of ALDH1+ cell population in Hep3B stable cell lines using an ALDFLUOR assay. The percentage of ALDH1+ cells in Hep3B cell lines was increased by the forced expression of TFF3 and decreased by depleted expression of TFF3 (Figure 4H).

Several stem cell markers such as *CD44, CD133 and ALDH1*, have been widely used to identify CSCs [50], and were observed to be upregulated in Hep3B-TFF3 cells but downregulated in Hep3B-siTFF3 cells as compared to their respective controls (Table 1). In addition, *FOXC2, SALL4*, *CSF1 and BMI1* mRNA levels were also increased in Hep3B-TFF3 cells, all of which have recently been suggested to be involved in promoting CSC-like characteristics in various cancer cells. *FOXC2* has been demonstrated not only to be involved in epithelial-mesenchymal transition (EMT) but also contributes to stem cell properties in cancer cells [51]. *SALL4* is a transcription factor and is responsible for regulating the stemness of hepatic CSCs [52]. Upregulation of CSF1 has been demonstrated to facilitate tumor invasion and contribute to CSC-like properties in HCC [53]. BMI1 has been found to regulate the self-renewal of hepatic cancer stem cells [54]. Taken together with the functional assays, the upregulation and downregulation of these stem cell-related genes in Hep3B-TFF3 cells and Hep3B-siTFF3 cells respectively, lend support to the notion that TFF3 regulates the stemness of Hep3B cells.

### TFF3 modulation of oncogenicity and CSC-like properties are BCL-2 dependent and mediated by AKT activation in HCC cells

**Figure 5:**
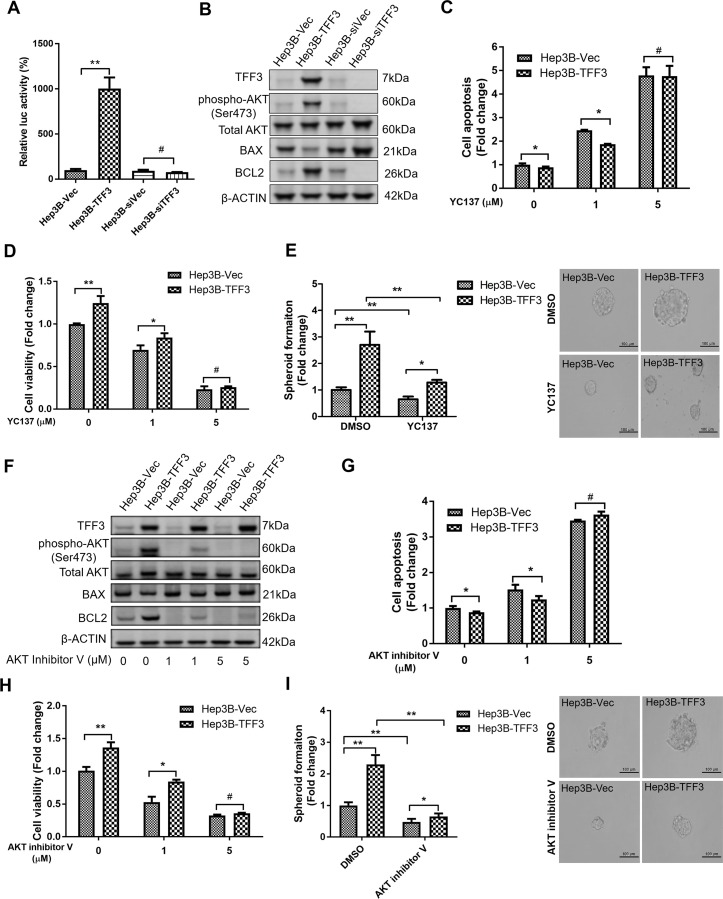
TFF3 promotes oncogenicity and CSC-like properties in a BCL-2 dependent manner that is mediated by AKT activation in Hep3B cells Hep3B cells were stably transfected with an expression vector containing the TFF3 gene (designated Hep3B-TFF3) or pIRESneo3 vector alone (Hep3B-Vec), TFF3 siRNA gene (designated Hep3B-siTFF3) or pSilencer vector alone (Hep3B-siVec). **(A)** TFF3 expression modulates BCL2 promoter activity. **(B)** Detection of protein expression using western blot, β-ACTIN was used as input control. **(C)** Cell apoptosis assay using Caspase 3/7 activity after YC137 treatment. **(D)** 3D Matrigel growth after YC137 treatment. Cell viability is shown in the histogram. **(E)** Spheroid formation after YC137 treatment. Number of spheroids is shown in the histogram. **(F)** Detection of protein expression after treatment of AKT inhibitor V, β-ACTIN was used as input control. **(G)** Cell apoptosis assay using Caspase 3/7 activity after treatment of AKT inhibitor V. **(H)** 3D Matrigel growth after AKT inhibitor V treatment. Cell viability is shown in the histogram. **(I)** Spheroid formation after treatment of AKT inhibitor V. Number of spheroids is shown in the histogram. Data were expressed as mean ±S.E.M. *, *p* < 0.05; **, *p* < 0.01; and ***, *p* < 0.001, *p* < 0.001; #, no significance.

Our previous study has suggested that TFF3 promotion of oncogenic behavior in breast cancer cells is BCL-2 dependent [16]. As *BCL-2* mRNA expression was also increased in HCC cells with forced expression of TFF3, TFF3 may partially execute its functional effects through BCL-2. Therefore, we further investigated whether TFF3 regulates BCL-2 expression in HCC cells. We observed that the *BCL-2* promoter luciferase reporter activity in Hep3B-TFF3 cells was greater than that in Hep3B-Vec cells (Figure 5A), indicating that TFF3 promotes *BCL-2* gene transcription in Hep3B cells. The ratio between the proapoptotic protein BAX and antiapoptotic protein BCL-2 was also significantly decreased as a result of forced expression of TFF3 in Hep3B cells (Table 1 and Figure 5B), which indicates a strong repression of the apoptotic cascade [55]. In contrast, depleted expression of TFF3 in HCC cells decreased BCL-2 protein expression and increased BAX protein expression (Figure 5B).

We next utilized the BCL-2 specific inhibitor YC137 to investigate the role of BCL-2 in TFF3-promoted HCC cell survival. The effect of BCL-2 inhibition on cell apoptosis was measured at different concentrations of YC137. YC137 at a concentration of 5μM fully abrogated the acquired cell survival advantage consequent to forced expression of TFF3 (Figure 5C), suggesting that TFF3-enhanced HCC cell survival is BCL-2-dependent. Moreover, YC137 at 5μM concentration fully abrogated the increased 3D Matrigel growth stimulated by forced expression of TFF3 in Hep3B-TFF3 cells (Figure 5D). Furthermore, the TFF3-promoted spheroid formation was partially abrogated by YC137 (Figure 5E). These results suggest that TFF3-stimulated oncogenicity and CSC-like properties are BCL-2-dependent.

The PI-3K/AKT signal transduction pathway is regulated by TFF3 [56]. We observed a significant increase in AKT activation (phosphorylation at Ser473) which correlated with increased BCL-2 expression in Hep3B-TFF3 cells as compared to Hep3B-Vec cells (Figure 5B). Hence, AKT activation may regulate BCL-2 expression in Hep3B cells in response to forced expression of TFF3. We therefore determined whether TFF3 modulates AKT activity in Hep3B cells. AKT inhibitor V was used to inhibit AKT activity [57]. AKT inhibitor V decreased both AKT activity and BCL-2 expression in a dose-dependent manner in Hep3B cells (Figure 5F). However, TFF3 expression was not affected by AKT inhibitor V treatment, suggesting that TFF3 is an upstream regulator of AKT activation in Hep3B cells. We also observed that cell apoptosis was significantly elevated after the addition of AKT inhibitor V in both Hep3B-Vec and Hep3B-TFF3 (Figure 5G). AKT inhibitor V at higher concentration (5μM) totally abrogated TFF3-enhanced cell survival. AKT inhibitor V at 5μM concentration also abrogated the increased 3D Matrigel growth stimulated by forced expression of TFF3 in Hep3B-TFF3 cells (Figure 5H). Hence, AKT activation is important for TFF3 promotion of cell survival and growth in Hep3B cells.

We further investigated whether TFF3 utilizes AKT to regulate CSC-like properties in Hep3B cells. AKT inhibitor V decreased spheroid formation in Hep3B cells (Figure 5I). As such, it can be deduced that TFF3 mediates oncogenicity and CSC-like behavior of HCC cells in an AKT-dependent manner.

### TFF3 promotes CSC-like behavior in doxorubicin resistant HCC cells

**Figure 6:**
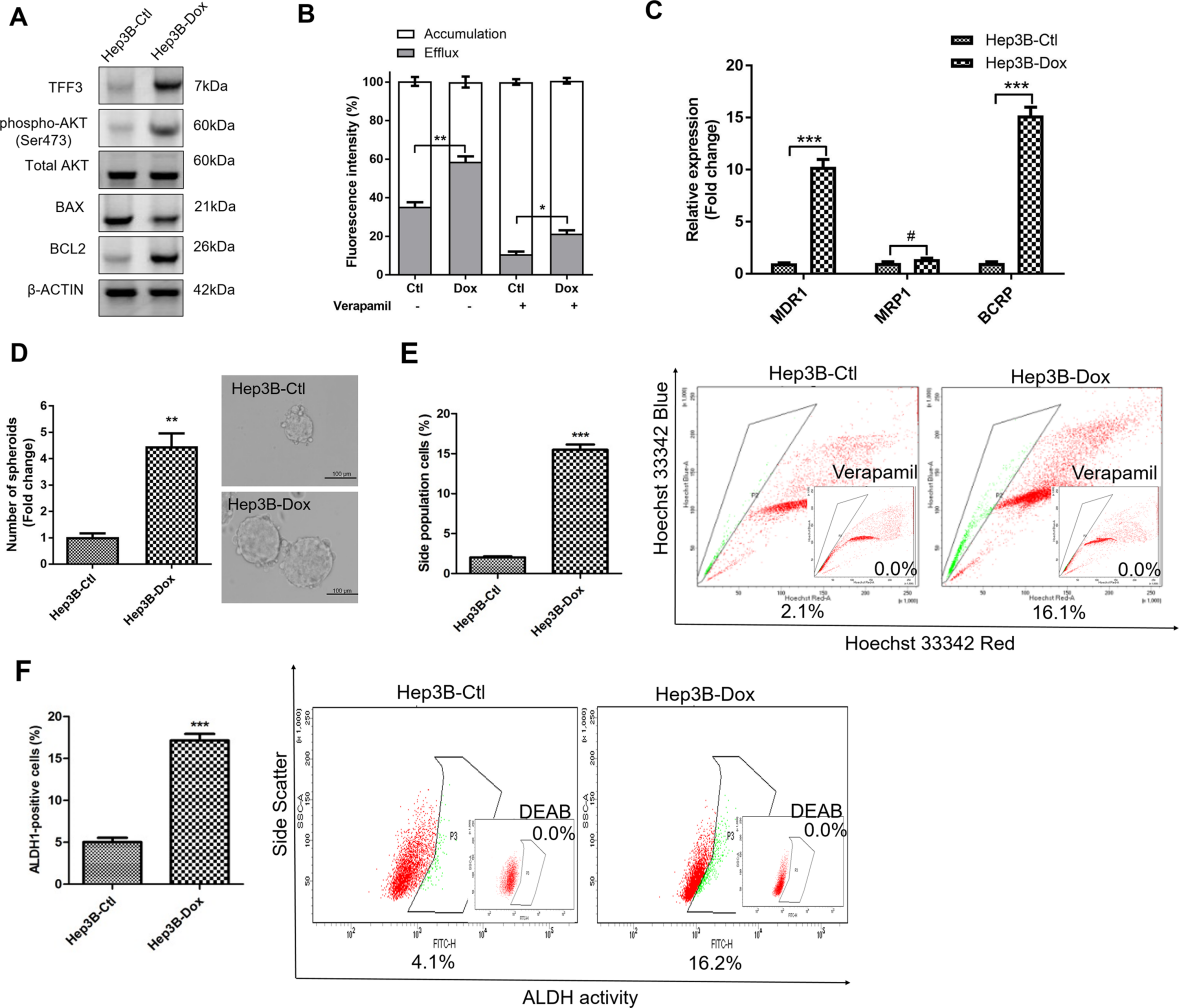
Doxorubicin resistant Hep3B cells exhibit increased TFF3 expression and CSC-like properties Resistant cells were maintained in media with IC_50_ concentration of doxorubicin (designated as Hep3B-Dox cells). The control cells during selection were cultured in medium containing DMSO (designated as Hep3B-Ctl cells). **(A)** Increase TFF3 expression was measured using western blot in Hep3B-Dox cells. **(B)** Efflux and accumulation of doxorubicin in Hep3B-Dox cells. **(C)** q-PCR analysis of drug resistant gene expression in Hep3B-Dox cells. **(D)** Spheroid formation. The number of spheroids formed by Hep3B-Ctl and –Dox cells is shown in the histogram. **(E)** The percentages of side-population in Hep3B-Ctl and –Dox cells were determined by flow cytometry. Verapamil was used to inhibit the ABC membrane transporter from extruding the Hoechst 33342 dye to establish the baseline for the identification of side population cells that efflux the Hoechst 33342 dye. **(F)** The percentages of ALDH+ cells in Hep3B-Ctl and –Dox cells were analysed using flow cytometry. The cells were incubated with the ALDEFLUOR substrate to define ALDH+ cells, while DEAB, a specific inhibitor for ALDH1A1 isoform, was used to establish the baseline fluorescence. Data were expressed as mean ±S.E.M. *, *p* < 0.05; **, *p* < 0.01; and ***, *p* < 0.001; #, no significance.

We further investigated the effect of TFF3 in modulating acquired doxorubicin resistance in HCC cells through the generation of Hep3B cells with acquired doxorubicin resistance. The response to doxorubicin was significantly decreased in Hep3B-Dox cells compared with Hep3B-Ctl cells (Supplementary Figure 3A). TFF3 expression, phosphorylated-AKT and BCL-2 expression were all elevated in Hep3B-Dox cells compared to Hep3B-Ctl cells (Figure 6A). Doxorubicin efflux was also enhanced in Hep3B-Dox cells compared to the control cells (Figure 6B). Furthermore, chemoresistance-associated gene expression was elevated in Hep3B-Dox cells, in which both MDR1 and BCRP were significantly increased in expression (Figure 6C). We further investigated whether CSC properties were increased in doxorubicin-resistant Hep3B cells. Spheroid formation ability, side population and the ALDH1+ cell population were increased in Hep3B-Dox cells compared with Hep3B-Ctl cells (Figures 6D-6F). Hence, HCC cells with acquired doxorubicin resistance exhibited increased TFF3 expression and enhanced CSC-like properties.

**Figure 7:**
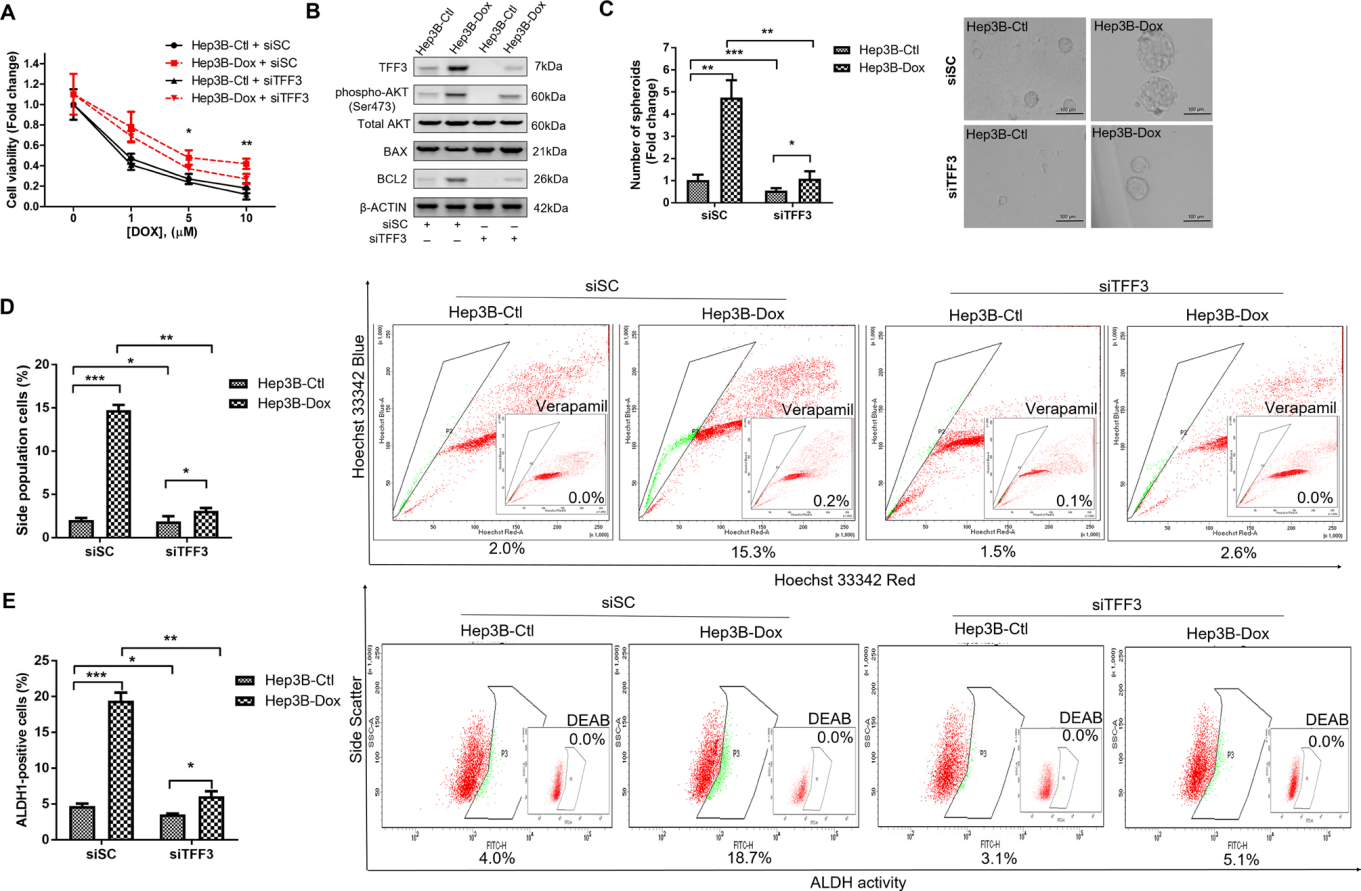
Depletion of TFF3 increases doxorubicin response and decreases CSC-like properties of Doxorubicin-resistant Hep3B cells **(A)** Chemosensitivity was determined in Hep3B-Dox with transient transfection of siTFF3. Cell viability was measured with AlamarBlue. **(B)** TFF3, AKT activation (Ser473) and BCL-2 expression in Hep3B-Dox cells were measured using western blot. **(C)** Spheroid formation. The number of spheroids formed by Hep3B-Ctl and –Dox cells ± siTFF3 is shown in the histogram. **(D)** The percentages of side-population in Hep3B-Ctl and –Dox cells ± siTFF3 were determined by flow cytometry. Verapamil was used to inhibit the ABC membrane transporter from extruding the Hoechst 33342 dye to establish the baseline for the identification of side population cells that efflux the Hoechst 33342 dye. **(E)** The percentages of ALDH+ cells in Hep3B-Ctl and –Dox cells ± siTFF3 were analysed using flow cytometry. The cells were incubated with the ALDEFLUOR substrate to define ALDH+ cells, while DEAB, a specific inhibitor for ALDH1A1 isoform, was used to establish the baseline fluorescence. Data were expressed as mean ±S.E.M. *, *p* < 0.05; **, *p* < 0.01; and ***, *p* < 0.001.

As TFF3 expression was increased in doxorubicin-resistant Hep3B cells, we further determined whether the increased TFF3 expression was responsible for the acquired resistance to doxorubicin. We observed that Hep3B-Dox cells were re-sensitized to doxorubicin by siRNA-mediated depletion of TFF3 (Figure 7A). Furthermore, AKT activity and BCL-2 expression were both attenuated after depletion of TFF3 in Hep3B-Dox cells (Figure 7B). Spheroid formation, side population and the percentage of ALDH1+ cells were also decreased in Hep3B-Dox cells after depletion of TFF3 (Figures 7C-7E). Hence, TFF3 exerts a functional role in maintaining CSC-like properties in doxorubicin-resistant Hep3B cells.

### AKT activation and BCL-2 expression are required for TFF3 stimulated CSC-like behavior in doxorubicin-resistant HCC cells

**Figure 8:**
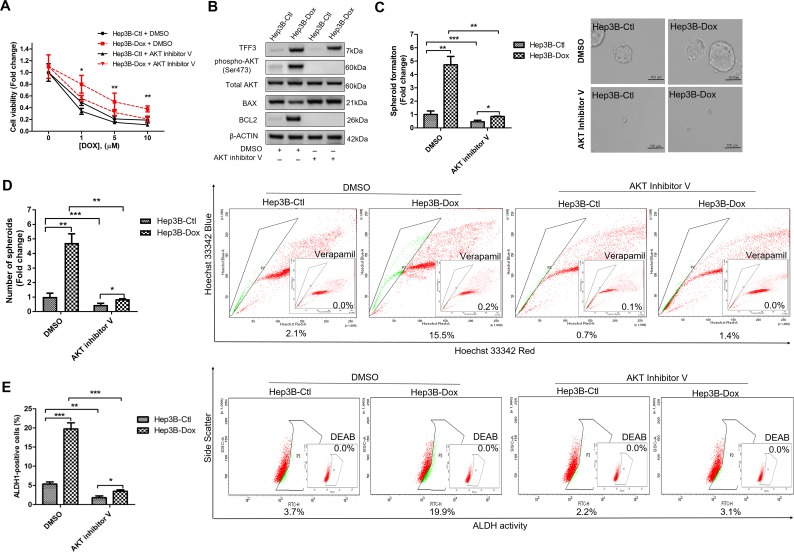
Inhibition of AKT increases doxorubicin response and decreases CSC-like properties of Doxorubicin-resistant Hep3B cells **(A)** Chemosensitivity was determined in Hep3B-Dox cells with AKT inhibitor V treatment. Cell viability was measured with alamarBlue. **(B)** TFF3, AKT activation (Ser473) and BCL-2 expression in Hep3B-Dox cells were measured using western blot. **(C)** Spheroid formation. The number of spheroids formed by Hep3B-Ctl and Hep3B-Dox cells, treated either with 5 μM AKT inhibitor V or DMSO vehicle, was shown in the histogram. **(D)** The percentages of side-population in Hep3B-Ctl and Hep3B-Dox cells, treated either with 5 μM AKT inhibitor V or DMSO vehicle, were analysed using flow cytometry. Verapamil was used to inhibit the ABC membrane transporter from extruding the Hoechst 33342 dye to establish the baseline for the identification of side population cells that efflux the Hoechst 33342 dye. **(E)** The percentages of ALDH+ cells in Hep3B-Ctl and Hep3B-Dox cells, treated either with 5 μM AKT inhibitor V or DMSO vehicle, were analysed using flow cytometry. The cells were incubated with the ALDEFLUOR substrate to define ALDH+ cells, while DEAB, a specific inhibitor for ALDH1A1 isoform, was used to establish the baseline fluorescence. Data were expressed as mean ±S.E.M. *, *p* < 0.05; **, *p* < 0.01; and ***, *p* < 0.001.

We further investigated whether AKT activity was required for mediating doxorubicin resistance in Hep3B cells. Hep3B-Dox cells could be re-sensitized to doxorubicin treatment by inhibiting AKT activity (Figure 8A). Furthermore, the AKT inhibitor V abrogated the increased BCL-2 expression in Hep3B-Dox cells (Figure 8B). In addition, spheroid formation ability, side population and percentage of ALDH1+ cells were dramatically reduced upon the inhibition of AKT activity in Hep3B-Dox cells (Figures 8C-8E). Moreover, the CSC-like behavior was also partially abrogated by BCL-2 inhibitor YC137 in Hep3B-Dox cells in terms of reduced spheroid formation, side population and the percentage of ALDH1+ cells (Supplementary Figures 3B-3D). These data suggest that TFF3-mediated doxorubicin resistance and CSC-like behavior in Hep3B cells is AKT- and BCL-2-dependent.

## DISCUSSION

Herein, we have demonstrated that TFF3 promotes oncogenicity of HCC cells. This is consistent with our previous studies demonstrating that TFF3 stimulates cell proliferation, survival, migration, and 3D and anchorage-independent cell growth of mammary and prostate carcinoma cells [16, 17, 20, 21]. Literature evidence also suggests a clinical correlation of TFF3 with HCC. Increased expression of TFF3 has previously been detected in HCC specimens and associated with tumor size and stage, providing evidence for the clinical significance of TFF3 expression in HCC [31, 32]. The increased expression of TFF3 in HCC was found to be associated with hypomethylation at CpG -260 of the *TFF3* promoter region [32]. Furthermore, the hypomethylated CpG sites locate into the binding motifs of several putative transcription factors in the TFF3 promoter region, indicating that TFF3 expression can be modulated by methylation status of the promoter region [58]. The expression levels of TFF3 gene in HCC tissues of HBx transgenic mice were also much higher than α-fetoprotein (AFP), suggesting that TFF3 is a more sensitive HCC biomarker than the conventional AFP [59]. Consistently, increased expression of TFF3 in HCC samples was observed in this study, which predicted for poorer survival outcome. Given the low TFF3 expression in normal liver tissue and elevated TFF3 expression functionally mediating oncogenic roles in HCC, TFF3 could be a potential biomarker and therapeutic target in HCC. However, elevated TFF3 expression is observed not only in HCC but also in other cancers [60] and inflammatory conditions [61]. Hence, it is suggested that a combination of multiple biomarkers, including TFF3, would improve the sensitivity and accuracy of HCC diagnosis.

Chemoresistance remains a major obstacle to the efficacy of chemotherapeutic treatment of HCC [3]. Doxorubicin is a commonly used chemotherapeutic agent in HCC [62]. However, doxorubicin treatment of patients with HCC results in poor tumor response rates of only approximately 10% [3]. In our study, increased TFF3 expression was observed to contribute to doxorubicin resistance in HCC cells and depletion of TFF3 re-sensitized HCC cells to doxorubicin. Consistently, TFF3 was reported to be upregulated in doxorubicin-treated liver tissue and hepatocytes [44]. This data is consistent with a previous study showing that the silencing of TFF3 in gastric cancer cells promotes sensitivity to doxorubicin [23]. In further support of our data herein, we have previously observed that TFF3 decreases sensitivity towards ionizing radiation in prostate cancer cells [20]. In addition, increased expression of TFF3 is fundamentally involved in acquired tamoxifen resistance and disease relapse in estrogen receptor positive (ER+) mammary carcinoma cells [16]. Similarly, increased TFF3 expression has also been observed to be associated with disease recurrence in ER+ patients treated with adjuvant aromatase inhibitor [22].

An important mechanism contributing to chemoresistance is evasion from apoptosis [63]. We have previously shown that TFF3 activates cell survival signaling pathways which contribute to anti-estrogen resistance in mammary carcinoma [16]. TFF3-enhanced cell survival and tamoxifen resistance have both been suggested to be dependent on BCL-2 [16]. Consistently in this study, we identified BCL-2 as an important downstream mediator of cell survival and doxorubicin resistance in HCC. The AKT pathway is a well-recognized mediator of cell survival in response to a number of stimuli [64]. AKT is known to transcriptionally upregulate BCL-2 through cAMP-response element-binding protein (CREB) [65]. Several studies have provided evidence for the activation of AKT by TFF3, which stimulates cell survival [25, 26]. In this study, TFF3-mediated cell survival has been shown to be dependent on AKT and BCL-2 in response to doxorubicin treatment. TFF3 has been reported to act through EGFR to activate several downstream signaling pathways, including AKT [66]. Moreover, in a recent study, we observed that forced expression of TFF3 in breast cancer cells increases c-Src activation [17]. c-Src is a non-receptor tyrosine kinase which mediates activation of signaling pathways to further regulate cell proliferation, invasion, survival, metastasis, and angiogenesis [67]. c-Src has also been demonstrated to increase AKT activation in HCC progression [68]. These findings imply that TFF3 may modulate AKT activation through c-Src. Further work is needed to elucidate the upstream mediators of TFF3 functions in HCC.

The presence of a CSC-like population has been associated with chemoresistance in cancers [69]. Hepatic CSCs can be identified by using Hoechst 33342 stain as a side population [70]. The side population cells exhibits an increased ability to efflux chemotherapeutic agents through several members of the ATP-binding cassette family, such as MDR1 and BCRP [71]. ALDH has been reported as a CSC marker and is positively correlated with CD133 expression [49]. We observed that TFF3 expression increased the CSC-like properties of HCC cells, concordant with the acquisition of doxorubicin resistance. Furthermore, accumulating evidence indicates that tumor cells undergoing EMT acquire the ability to enter a CSC-like state, wherein they exhibit enhanced self-renewal properties, increased oncogenic potential and enhanced chemoresistance [72]. The poor prognosis in late stage HCC including chemoresistance, invasive dissemination and tumor relapse, are attributed to the EMT and CSC characteristics [72]. The link between EMT and CSC has been well-established in various cancers, including HCC [73]. In this study, several EMT and stem cell-related genes were also modulated by forced or depleted expression of TFF3. Consistently, TFF3 has previously been shown to promote migration and invasion of breast cancer cells, and modulate the expression of epithelial, mesenchymal and metastatic gene markers [17]. Increasing evidence has supported the role of the PI3K/AKT/mTOR signaling pathway in the maintenance of CSC in several cancers [74]. AKT has recently been reported to be a critical regulator of CSC survival and maintenance of the CSC phenotype in breast cancer [75 Importantly, it was reported that the activation of the AKT and BCL-2 cell survival response in CSCs confer chemoresistance in HCC [10]. In addition, AKT activation has been demonstrated to be involved in the regulation of ABC transporters and ALDH expression in HCC cells [76, 77]. BCL-2, as one of the downstream effectors of the AKT pathway, has also been implicated in chemotherapy resistance in CSCs [78]. Our findings suggest that TFF3-stimulated CSC functions, contributing to doxorubicin resistance, are regulated by AKT-dependent expression of BCL-2.

Taken together, TFF3 increases oncogenicity of HCC cells and increased TFF3 expression is associated with poorer survival outcome in HCC. Importantly, TFF3 reduces the sensitivity of HCC cells to doxorubicin and mediates acquired doxorubicin resistance through increasing survival, drug efflux and promoting CSC-like properties in HCC cells. Based on the current findings, TFF3 is a potential biomarker and therapeutic target in HCC. In addition, inhibition of TFF3 could be a potential approach to abrogate the development of chemoresistance in HCC and warrants investigation of the clinical potential of the combination of doxorubicin with a TFF3 inhibitor.

## MATERIALS AND METHODS

### Histopathological analysis

A cohort of 150 HCC patients was enrolled in this study with informed patient consent in accordance with the Declaration of Helsinki. The institutional review board of Anhui Medical University (AMU) (Hefei, Anhui, People's Republic of China) approved the protocol for the use of patient specimens in this study. Formalin-fixed and paraffin-embedded HCC tissue specimens (n = 138) and adjacent non-tumor specimens (n = 110) were studied. All patients underwent surgery at the First Affiliated Hospital of AMU. The immunohistochemical analysis of TFF3 protein expression was performed on 4 μm thick TMA sections with rabbit monoclonal antibodies against TFF3 (ab108599, 1:100, Abcam) by DAKO EnVison detection system containing DAKO EnVision + ™ and DAB + chromogenic substrate (DAKO, Denmark). The details of the cohort and IHC scoring methodology have previously been described [79]. Briefly, we defined the evaluation of IHC staining was based on the combined expression pattern of all of the tissue from each patient sample. Stained sections were independently assessed for expression of TFF3 with a light microscope by two pathologists without knowledge of the samples associated clinicopathologic information. The sections were scored on the basis of the percentage of cells with staining relative to the background and the staining intensity. Firstly, the extent of staining was scored as 1 (< 33%), 2 (33%-67%), and 3 (> 67%) according to the percentage of the positive staining areas and staining intensity was scored as 1 (no stain or weak), 2 (medium), and 3 (strong). The sum of the extent and intensity score was used as the staining score (1-6) for TFF3 expression. Scores of ≤ 3 was designated as negative expression and ≥ 4 was designated as positive expression. Furthermore, a statistical correlation of TFF3 expression in HCC with the clinicopathological parameters of HCC patients was analysed using the Spearman rank correlation according to procedures previously described [80].

### Cell culture and transfection

The human hepatocellular carcinoma cell lines Huh7, Hep3B, HepG2, and PLC\PRF\5 were originally obtained from the American Type Culture Collection (Rockville, MD, USA). H2P and H2M cell lines were kindly provided by Dr. Chen from Cancer Science Institute of Singapore. Normal hepatocyte cell line LO2 was kindly provided by Institute of Biochemistry and Cell Biology, Chinese Academy of Sciences. All cell lines were cultured in DMEM (Hyclone, USA) supplemented with 10% heat-inactivated fetal bovine serum (FBS) (Hyclone, USA), 100 IU/mL penicillin and 100 μg/mL streptomycin (Invitrogen, USA) as recommended.

Huh7, Hep3B and HepG2 cells with forced expression of TFF3 or siRNA-mediated depletion of TFF3 were transfected with FuGENE 6 transfection reagent (Promega, USA) as previously described [16]. Hep3B and Huh7 cells were transfected with pIRES-TFF3 or the empty vector (control). After G418 selection, the stable cell lines were obtained and designated as Hep3B-TFF3 and Hep3B-Vec or Huh7-TFF3 and Huh7-Vec respectively. Hep3B and HepG2 cells were transfected with pSilencer-siTFF3 or the empty vector (control). After Hygromycin B selection, the stable cell lines were obtained and designated as Hep3B-siTFF3 and Hep3B-siVec or HepG2-siTFF3 and HepG2-siVec respectively. Serum free media was mixed with FuGENE 6 transfection reagent and the respective plasmids for HCC cell transfection. After that, cell culture media was changed into selection media containing selection antibiotics. Selection media was replaced every 3 days until the formation of positive colonies was observed. After 4 weeks of selection, pooled stable transfectants were collected and expanded for cell function assays.

### mRNA and protein expression analysis

Total RNA was isolated from exponentially growing cells (70% confluence) using RNeasy mini Kit (Qiagen, Netherlands) according to by manufacturer's instruction. Real-time quantitative PCR (qPCR) was also performed to analyse mRNA expression levels [16]. Primer used for RT-PCR and qPCR are as previously described [81]. Protein expression was analysed using western blot by using the following antibodies: TFF3 (ab108599, 1:1000, Abcam); phospho-AKT1 S473 (ab66138,1:2000, Abcam); pan-AKT (ab8805, 1:2000, Abcam); β-Actin (sc-47778, 1:10000, Santa Cruz), BCL-2 (sc-509, 1:5000, Santa Cruz), Bax (sc-70407, 1:5000, Santa Cruz). The secondary anti-rabbit and anti-mouse horseradish peroxidase (HRP)-conjugated antibodies were purchased from Cell Signaling Technology. Proteins were visualized using the chemiluminescence ECL kit (SuperSignal West Pico substrate; Pierce, Rockford, IL) and read on ImageQuant system LAS500 (GE Healthcare).

### Oncogenicity assays

Cell proliferation was measured using total cell number, bromodeoxyuridine (BrdU) incorporation, and cell cycle analysis as previously described [16]. Apoptotic cell death was determined using Hoechst 33342 staining and Caspase-Glo caspase 3/7 kit (Promega) according to recommended protocols. Foci formation, colony formation in soft agar assay, three-dimensional Matrigel assay, transwell migration and invasion assays were performed as previously described [82]. Details of the experimental procedure can be found in Supplementary Material and Methods. All images were taken using bright field microscopy (Olympus, Tokyo, Japan).

### Generation of chemoresistance cells

The cytotoxic effects of chemotherapy drugs on the stable cells were measured using MTT assay. In a 96-well plate, 5 × 10^3^ cells were seeded per well and allowed to adhere overnight. After that, 200 μl of media containing doxorubicin at different concentrations were added to the wells. After 72 h incubation, media was changed and MTT reagent was added to each well. The absorbance (450 nm and 650 nm) was measured using a microplate reader (Tecan, Infinite 200). The relative cell survival (%). measured by MTT was calculated by comparing the absorbance values (450 nm) of treated cells to untreated cells. The IC_50_ values of doxorubicin in the stable cells were analysed using nonlinear regression in GraphPad Prism 5.

To generate doxorubicin resistant cells, Hep3B cells were grown under selective pressure (IC_50_) for 3 days and subsequently for 3 days without doxorubicin. This cycle was repeated until we observed significant doxorubicin resistance in doxorubicin-treated Hep3B cells by assessing cell viability as previously described [80]. Resistant cells were maintained in media with IC_50_ concentration of doxorubicin (designated as Hep3B-Dox cells). The control cells during selection were cultured in medium containing DMSO (designated as Hep3B-Ctl cells).

### Doxorubicin efflux and accumulation assays

Doxorubicin efflux and accumulation assays were performed as previously described [83]. Cells were seeded in 24-well plates at 5 ×10^5^ cells per well until cell density reached 80% confluence. After that, cells were treated with 1 μM doxorubicin for 2 hours and washed with PBS twice to remove doxorubicin. Cells were then incubated with doxorubicin-free media for up to 120 minutes. Subsequently, the conditioned media were collected for analyzing doxorubicin efflux, and cell pellets were used to measure doxorubicin accumulation. The fluorescence intensities of both intracellularly accumulated and effluxed doxorubicin were measured using an excitation wavelength of 490 nm and emission wavelength of 570 nm using a fluorescence microplate reader (Tecan, Infinite 200).

### Spheroid formation assay

Cells were trypsinized and washed with serum free media. Harvested cells were filtered by cell strainer (40 μm) to generate single cells, which were then suspended in serum-free cancer stem cell growth media. The CSC growth media used consists of serum-free DMEM/F12 (Hyclone) containing penicillin-streptomycin (Bio-west), 10 ng/ml recombinant human basic FGF (BD Biosciences), 20 ng/ml recombinant human EGF (Sigma-Aldrich), 2% B27 supplements (Gibco), 1% N2 supplement (Gibco) and 5 μg/ml bovine insulin (Sigma-Aldrich). Cells were then plated in ultra-low attachment 6-well plates at a density of 5,000 cells/well or in ultra-low attachment 96-well plates at a density of 10 cells/well. Spheroid formation were observed and counted under microscope after 7 days in culture [84].

### ALDEFLUOR assay and Hoechst 33342 side population assay

The aldehyde dehydrogenase-positive cell population was measured using ALDEFLUOR kit (STEMCELL Technologies) according to the provided protocol. For side population assay, cells were trypsinized and collected by centrifugation at 200 × g for 5 minutes. The collected cells were incubated in DMEM media containing 2% FBS and 5 μg/ml Hoechst 33342 for 90 minutes at 37°C. For control samples, ABC transporter inhibitor verapamil (Sigma-Aldrich) was added to cells at a final concentration of 50 μM. After 30 minutes of incubation, cells were centrifuged at 200 × g for 5 minutes and suspended in 1 ml cold PBS. Cells were filtered through 40 μm cell strainers to generate single-cell suspension for FACS analysis using FACS LSR II.

### Tumor xenograft analysis

Xenograft study was performed in accordance with a protocol approved by animal care and ethics committee of University of Science and Technology of China and that conformed to the legal mandate and national guidelines for the care and maintenance of laboratory animals. Hep3B-Vec, Hep3B-TFF3, Hep3B-siVec and Hep3B-siTFF3 cells (5 × 10^6^) were injected subcutaneously into the left or right flanks of immunodeficient nude mice (Slac Laboratory Co, Shanghai, China). Each group consisted of six nude mice. The tumor volumes were measured every 3-4 days after injection for 1 week. Tumor volume growth was measured over time by digital calipers, and the tumor volume in mm3 is calculated by the formula: Volume = (width)^2^ × length/2. After six weeks, mice were sacrificed by CO_2_ inhalation. The tumor tissues were resected for immunohistochemistry studies as described earlier [79].

### Statistics

Spearman rank correlation was used to test the association between TFF3 expression and clinicopathological features of HCC patients. The relationship of TFF3 expression and the overall survival or relapse free survival of HCC patients were analysed by Kaplan-Meier analyses, and the log-rank test was performed to analyse the statistical significance. All experiments were repeated at least 3 times. All numerical data were expressed as mean ±S.E.M. and statistical significance was assessed by Student's t-test (P<0.05 was considered as significant) using Microsoft Excel unless otherwise indicated.

## SUPPLEMENTARY MATERIALS AND METHODS AND FIGURES


