# Supplementary material for: Trefoil factor 3 mediation of oncogenicity and chemoresistance in hepatocellular carcinoma is AKT-BCL-2 dependent
Source: Oncotarget. 2017 Apr 7;8(24):39323–44. doi: 10.18632/oncotarget.16950 (PMC5503616; doi:10.18632/oncotarget.16950)
Supplement: Supplementary file 1 [file oncotarget-08-39323-s001.pdf]

Trefoil factor 3 mediation of oncogenicity and chemoresistance in hepatocellular carcinoma is AKT-BCL-2 dependent

SUPPLEMENTARY MATERIALS AND METHODS

Total cell number counting

Cells were plated into 6-well cell culture plate at 5×10^4 cells/well and cultured for 8 h at 37°C in incubator to ensure that they attached to the culture surface. Cell culture media was then changed into either 0.2% FBS or 10% FBS media. Assays were set up in triplicate and cells were counted every 2-3 days during culture. On indicated days, cells in each well were trypsinised with 0.5% trypsin and harvested by centrifuging at 200 × g for 5 minutes. Finally, collected cells were counted using a haemocytometer.

Cell viability assay

Cells were plated into 96-well cell culture plate at 10^4 cells/well and cultured at 37°C in incubator. MTT assay was conducted to measure cell viability. Regarding drug treatments, drugs were dissolved in DMSO and added in the serum free media to the final working concentration. Plates were incubated for 3 days and cell viability were measure with MTT assay. DMSO without drugs were made as control for drug treatment assays.

BrdU incorporation assay

BrdU incorporation assay was carried out using BrdU cell proliferation assay following the provided protocol (Merck Millipore). One hundred µl of cells at 10^5 cells/ml were seeded into a 96-well culture plate. After cell attached the cell culture plate, 20 µl of fresh culture media containing BrdU (dilute 1: 2000) were added to each well and incubated 24 hours. After that, media were removed and dry the plate with towel papers. When plates were dry, 200 µl of denaturing solution were pipetted to each well and incubated 30 minutes at room temperature. Subsequently, liquid were removed and 100 µl of anti-BrdU antibody (dilute 1: 100 in antibody buffer) were added to each well and incubate for 1 hour at ambient temperature. Removed anti-BrdU antibody and wash with washing buffer three times. After that, Goat Anti-Mouse IgG HRP conjugate in the conjugate diluent were added into each well and incubate for 30 minutes at room temperature. After removal of content in the wells, washed wells 3 times, 100 µl of substrate solution were added to each well and incubate at room temperature for 15 min. After incubation, 100 µl of stop solution were added and absorbance were measured using micro-plate reader at 450-540 nm.

Cell cycle analysis

Cultured cells after treatment were trypsinized and washed in PBS. Harvested cells were fixed in cold 70% ethanol for 30 min at 4°C. Fixed cells were washed with PBS twice and centrifuged to collect cells. After that, cells were at incubated with 1% Triton X-100/PBS solution and treated with 5 µl of RNase (1 mg/ml). Finally, 200 µl PI (50 µg/ml) was added into tube and incubated for 15 minutes at RT before being assessed by BD FACSAriaII flow cytometer (BD Biosciences).

Cell apoptosis measurement

Cell nucleus was stained with Hoechst 33342 to analyze apoptotic nucleus under fluorescent microscopic. Cells were cultured in serum free media to induce apoptosis. After 24 hour or 48 hour, the cells were fixed in fixing buffer (PBS containing 4% paraformaldehyde and 1% Triton-X-100) and stained with Hoeschst 33342 in PBS for 15 minutes at ambient temperature. Apoptotic nuclear was discriminated from viable cells according to their nuclear morphology (condensed and fragmented nuclear with high intensity of the blue fluorescence). In order to statistical analyse the apoptosis rate, 200 cells were counted in 8 random microscopic fields at ×400 magnification.

Apoptotic cell death was also determined using Caspase-Glo caspase 3/7 kit (Promega) following the recommended protocols. Cells were plated and cultured in white-walled 96-well plates at a density of 5×10^4 cells/well. After starvation or drug treatment-induced apoptosis, added Caspase-Glo 3/7 reagent (100µl) to white-walled 96-well plate. Cell plates were gently mixed using a plate shaker at 300–500 rpm for 30 seconds and incubate at room temperature for 1 hour. Finally, luminescence of each sample were measured with a fluorescence/luminesence microplate reader (Tecan).

Anchorage-independent growth

Soft agar colony formation was performed to investigate anchorage-independent growth of HCC cells. Each well of a 96 well plate was covered with a base agar

layer of 0.5% agarose in serum free media. After that, 1×10^5 cells suspended in 0.35% agarose and 5% FBS was added to each well on the top of the base agarose. After two weeks of culture, cells were stained with MTT and observed under microscopy. For the assay measuring colony formation after chemotherapeutic drug treatments, cells were treated with drug for 24h prior to embedding in agarose. The media was changed every second day for 14 days. Each experiment was performed in triplicate. The total number of colonies in each well was counted at 40 × magnification (Olympus).

Three-dimensional culture assay

Growth factor reduced Matrigel was used in 3D cell culture. First, Matrigel (50µl/well for 96-well plate) was added to coat the surface of cell culture plate. After that, Matrigel coated plates were placed in cell culture incubator for 30 minutes to allow gelation. Meanwhile, cells were trypsinised and harvested by centrifuging at 200 × g for 5 min. Subsequently, cells were then suspended in media and filtered through 40 µm cell strainer to obtain a single cell suspension. One thousand cells were seeded into the coating 96-well plate. Two hundred µl Matrigel (4%) in 5% serum media were pipetted onto the solidified Matrigel in each well. Cells were cultured in a 5% CO_2 humidified incubator at 37°C for 12 days. Every second day, 100µl waste media were removed and 100µl of fresh Matrigel (4%) in 5% serum media were added to the wells. For assays involving drug treatments, the 4% Matrigel solution containing Doxorubicin or 5-FU was added to the cells on day 3. After 10 day growth, 20 µl of AlamarBlue was added to each well, and left for 4 hours in the CO_2 incubator. After incubation, the plate was immediately read spectrofluorometrically on 560nm/590nm using microplate reader (Tecan).

Migration and invasion assay

In vitro cell migration and invasion assays were performed using 24-well cell invasion chamber (8 µm pore size) following the provided protocol (BD Biosciences). For invasion assessment, inserts were coated with 10% Matrigel before seeding the cells. Briefly, cells were seeded (2.5×10^4 cells) with serum free media in the upper chamber and complete media were added in the bottom well. After 48h incubation, the media in both upper and lower chamber were removed. Cells on the lower surface of the inserts were washed with PBS and fixed with 4% paraformaldehyde. After removed the non-migrating or non-invading cells on the upper surface of the inserts with cotton tipped swab, the cells on lower surface of inserts were stained with Hoechst 33342 (Sigma). The migrated and invaded cells were observed and counted using fluorescence microscope.

SUPPLEMENTARY FIGURES

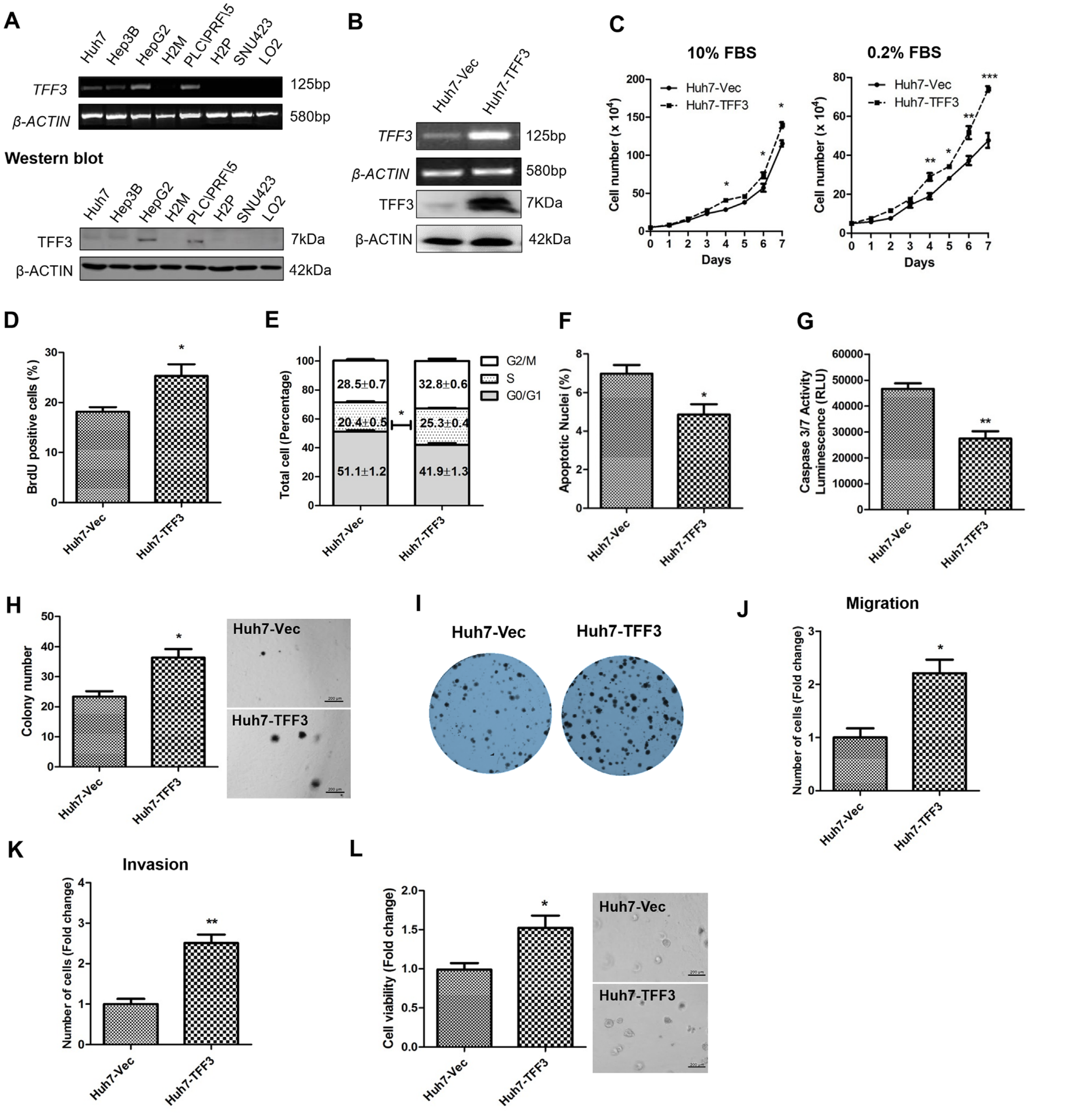

Supplementary Figure 1: Forced expression of TFF3 in HCC cells increases oncogenicity in HCC cells. **(A)** Detection of TFF3 expression in different HCC cell lines. **(B)** Detection of forced expression of TFF3 in Huh7 stable cells. **(C)** Total cell count in DMEM media supplemented with 10% or 0.2% FBS over 7 days. **(D)** BrdU incorporation assay. **(E)** Cell cycle analysis. **(F)** Apoptosis assay. Percentage of the apoptic cell after 24h serum starvation. **(G)** Caspase 3/7 activity after 24h serum starvation. **(H)** Soft agar colony formation. Colony numbers are shown in the histogram. **(I)** Foci formation. **(J)** Cell migration assay. **(K)** Cell invasion assay. Number of cells penetrating the transwell membrane. **(L)** 3D Matrigel growth. Cell viability is shown in the histogram. Data were expressed as mean ±S.E.M. *, $p < 0.05$; **, $p < 0.01$; and ***, $p < 0.001$.

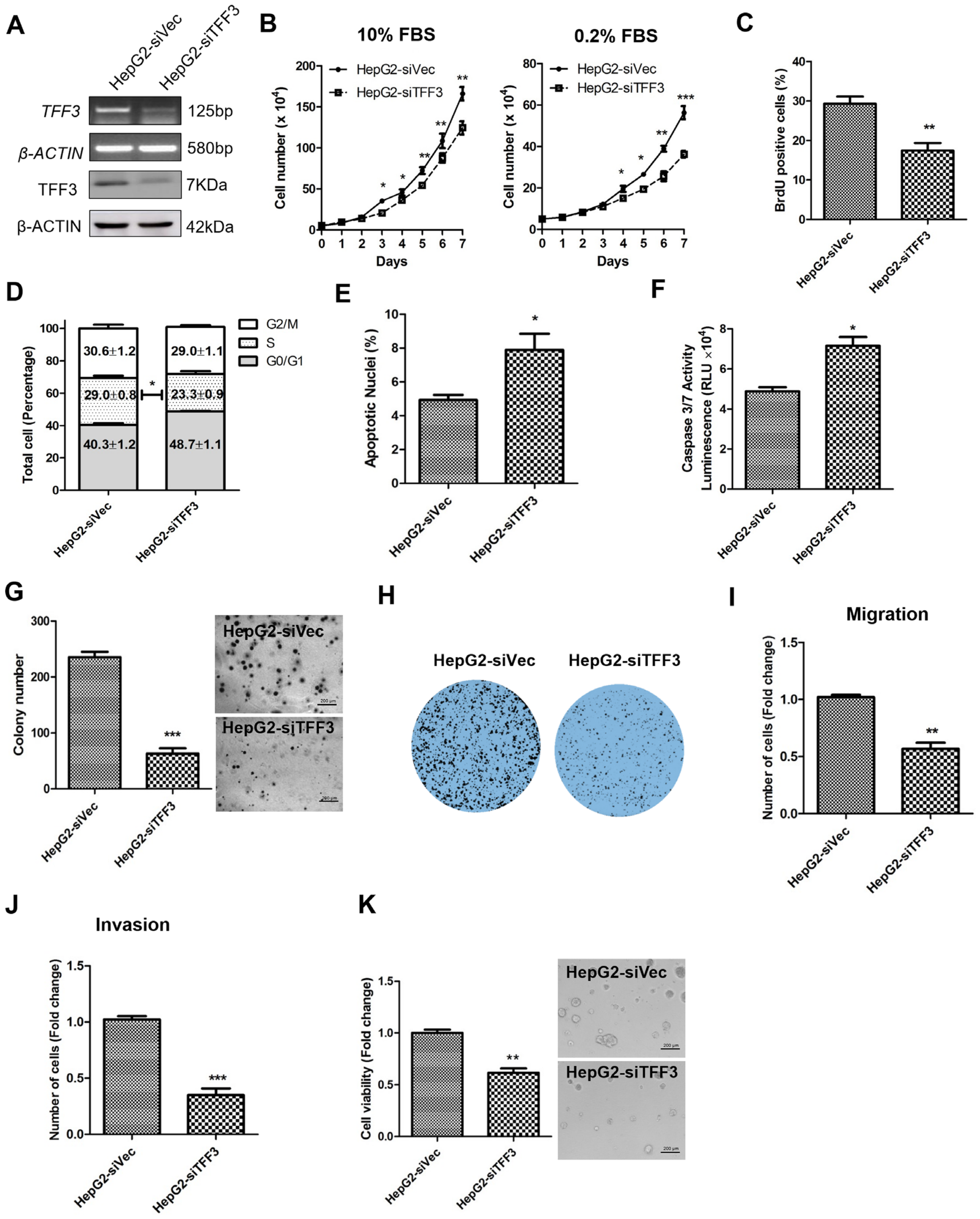

Supplementary Figure 2: Depleted expression of TFF3 decreases oncogenicity in HepG2 cells. (A) Detection of forced expression of TFF3 in HepG2 stable cells. **(B)** Total cell count in DMEM media supplemented with 10% or 0.2% FBS over 7 days. **(C)** BrdU incorporation assay. **(D)** Cell cycle analysis. **(E)** Apoptosis assay. Percentage of the apoptic cell after 24h serum starvation. **(F)** Caspase 3/7 activity after 24h serum starvation. **(G)** Soft agar colony formation. Colony numbers are shown in the histogram. **(H)** Foci formation. **(I)** Cell migration assay. **(J)** Cell invasion assay. Number of cells penetrating the transwell membrane. **(K)** 3D Matrigel growth. Cell viability is shown in the histogram. Data were expressed as mean ±S.E.M. *, $p < 0.05$; **, $p < 0.01$; and ***, $p < 0.001$.

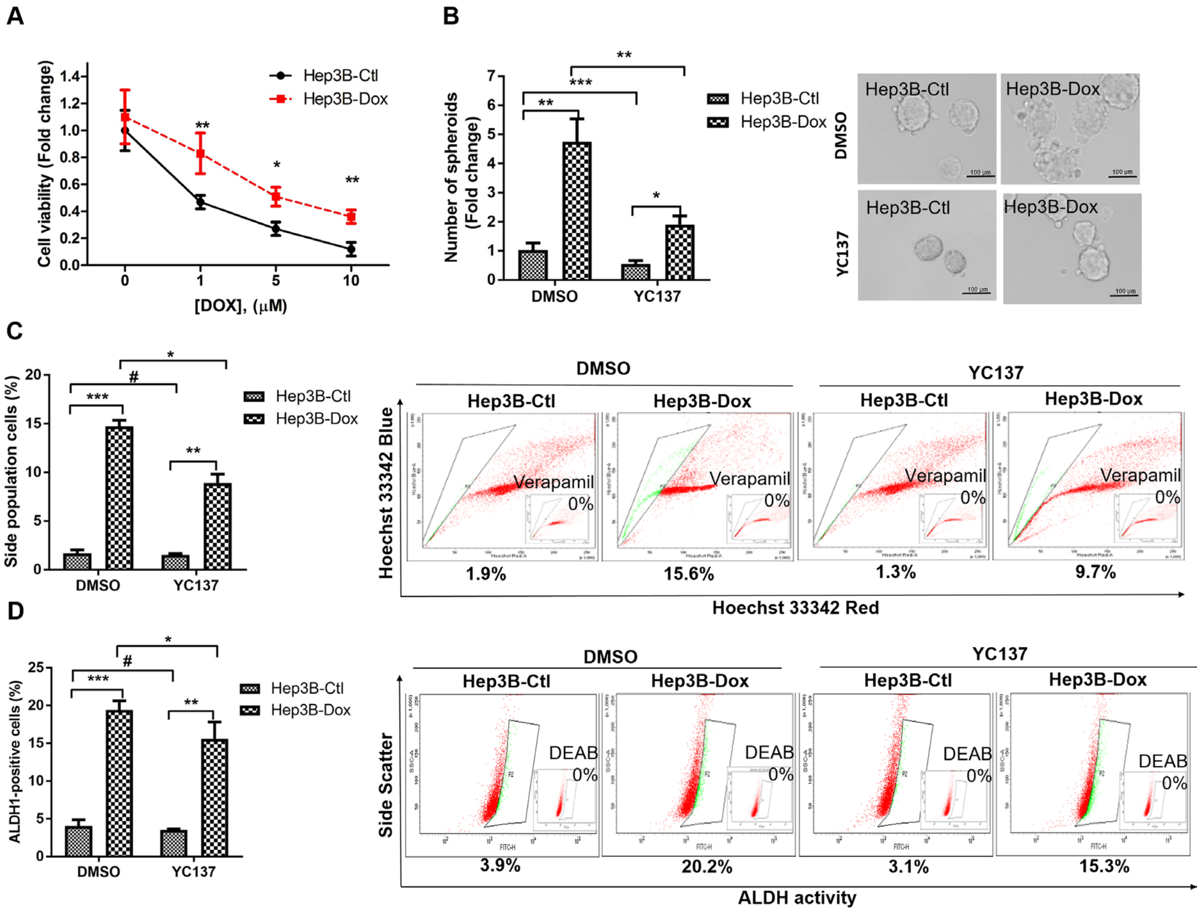

Supplementary Figure 3: Inhibition of BCL2 increases doxorubicin response and decreases CSC-like properties of Doxorubicin-resistant Hep3B cells. **(A)** Chemosensitivity in Hep3B-Dox cells was determined with YC137 treatment. Cell viability was measured with alamarBlue. **(B)** Spheroid formation. Number of spheroids were shown in the histogram. **(C)** Percentage of side-population cell was analysed using flow cytometry. **(D)** Percentage of ALDH+ cell was analysed by flow cytometry. Data were expressed as mean ±S.E.M. *, $p < 0.05$; **, $p < 0.01$; and ***, $p < 0.001$; #, no significance.
